# Pembrolizumab plus axitinib versus sunitinib for advanced clear cell renal cell carcinoma: 5-year survival and biomarker analyses of the phase 3 KEYNOTE-426 trial

**DOI:** 10.1038/s41591-025-03867-5

**Published:** 2025-08-01

**Authors:** Brian I. Rini, Elizabeth R. Plimack, Viktor Stus, Rustem Gafanov, Tom Waddell, Dmitry Nosov, Frédéric Pouliot, Boris Alekseev, Denis Soulières, Bohuslav Melichar, Ihor Vynnychenko, Sergio Jobim de Azevedo, Delphine Borchiellini, Raymond S. McDermott, Jens Bedke, Satoshi Tamada, Sterling Wu, Julia Markensohn, Yiwei Zhang, Andrey Loboda, Amir Vajdi, Rodolfo F. Perini, Joseph Burgents, Thomas Powles

**Affiliations:** 1 Vanderbilt-Ingram Cancer Center, Nashville, TN USA; 2 Fox Chase Cancer Center, Philadelphia, PA USA; 3 Dnipro State Medical University, Dnipro, Ukraine; 4 Russian Scientific Center of Roentgenoradiology, Moscow, Russia; 5 The Christie NHS Foundation Trust, Manchester, UK; 6 Central Clinical Hospital With Outpatient Clinic, Moscow, Russia; 7 CHU of Québec and Laval University, Quebec, Quebec Canada; 8 P. Herzen Moscow Oncology Research Institute, Ministry of Health of the Russian Federation, Moscow, Russia; 9 Centre Hospitalier de l’Université de Montréal, Montreal, Quebec Canada; 10 Palacký University Medical School and Teaching Hospital, Olomouc, Czech Republic; 11 Sumy State University, Sumy Regional Oncology Center, Sumy, Ukraine; 12 Hospital de Clínicas de Porto Alegre, Porto Alegre, Brazil; 13 Centre Antoine Lacassagne, Université Côte d’Azur, Nice, France; 14 St. Vincentʼs University Hospital and University College Dublin, Dublin, Ireland; 15 Department of Urology and Transplantation Surgery, Eva Mayr-Stihl Cancer Center, Klinikum Stuttgart, Stuttgart, Germany; 16 Bell-Land General Hospital, Osaka, Japan; 17 Merck & Co., Inc., Rahway, NJ USA; 18 Barts Health Biomedical Research Center, Queen Mary’s University of London ECMC, London, UK

**Keywords:** Predictive markers, Cancer immunotherapy

## Abstract

At the first interim analysis of the phase 3 KEYNOTE-426 trial, first-line pembrolizumab plus axitinib showed superior overall survival (OS), progression-free survival (PFS) and objective response rate (ORR) over sunitinib for advanced renal cell carcinoma (RCC). To assess long-term durability of clinical outcomes and elucidate predictive biomarkers for RCC, we performed efficacy and prespecified exploratory biomarker analyses from KEYNOTE-426 with ≥5 years of follow-up. Pembrolizumab plus axitinib showed sustained benefits in OS (hazard ratio: 0.84; 95% confidence interval: 0.71–0.99), PFS (hazard ratio: 0.69; 95% confidence interval: 0.59–0.81) and ORR (60.6% versus 39.6%) compared to sunitinib. An 18-gene T-cell-inflamed gene expression profile (Tcell_inf_GEP) was positively associated with OS (*P* = 0.002), PFS (*P* < 0.0001) and ORR (*P* < 0.0001) within the pembrolizumab plus axitinib arm. An angiogenesis signature was positively associated with OS (*P* = 0.004) within the pembrolizumab plus axitinib arm and with OS (*P* < 0.0001), PFS (*P* < 0.001) and ORR (*P* = 0.002) within the sunitinib arm. Across arms, programmed cell death ligand 1 combined positive score was only associated (negatively) with OS within the sunitinib arm (*P* = 0.025). Additionally, *PBRM1* (polybromo-1) mutation had a positive association with ORR (*P* = 0.002) within the pembrolizumab plus axitinib arm. Within the sunitinib arm, OS was positively associated with *VHL* (von Hippel–Lindau tumor suppressor gene) (*P* = 0.040) and *PBRM1* (*P* = 0.010) mutations and was negatively associated with *BAP1* (*BRCA1-*associated protein 1) mutation (*P* = 0.019). Results showed a sustained clinical benefit with pembrolizumab plus axitinib over sunitinib and provide valuable information on biomarkers for immunotherapy-based treatment combinations in advanced RCC. Prospective clinical investigations are needed for biomarker-directed treatment for advanced RCC. ClinicalTrials.gov identifier: NCT02853331.

## Abstract (web summary)

Five-year follow-up and prespecified exploratory biomarker analysis from the phase 3 KEYNOTE-426 trial show that pembrolizumab plus axitinib compared to sunitinib as first-line therapy continues to show overall and progression-free survival benefits in patients with advanced clear cell renal cell carcinoma and indicate that a T-cell-inflamed gene expression profile is predictive of response.

## Main

The combination of the programmed cell death protein 1 (PD-1) inhibitor pembrolizumab and the vascular endothelial growth factor receptor (VEGFR) tyrosine kinase inhibitor (TKI) axitinib is standard-of-care first-line treatment for patients with advanced clear cell RCC, as a result of outcomes from the open-label, randomized, phase 3 KEYNOTE-426 trial^1–5^. KEYNOTE-426 was the first trial of a PD-1 inhibitor and VEGFR-TKI combination in the first-line treatment setting and, therefore, has the longest follow-up duration of any combination of a PD-1 or programmed cell death ligand 1 (PD-L1) inhibitor and a TKI. Because the treatment duration of pembrolizumab is limited to 2 years, it is important to assess the long-term durability of clinical outcomes.

There is an unmet need for biomarkers that are predictive of patient outcomes after using available first-line treatment options in RCC^6,7^. Studies to evaluate predictive and prognostic biomarkers in metastatic RCC have been largely derived from phase 3 studies (for example, IMmotion151, JAVELIN Renal 101 and CheckMate 9ER) in which similar mechanisms (for example, PD-L1 inhibitor plus VEGF-TKI) but different drugs were evaluated as frontline therapy^8–22^. Thus, extrapolation of these data to define predictive biomarkers in frontline therapy for advanced RCC is potentially confounded. Nonetheless, the studies yielded relevant biological insight into the role of molecular features of RCC, including PD-L1 expression, interferon gamma (IFNγ) RNA signatures, specific DNA alterations (such as *PBRM1* mutations), mRNA-based molecular clusters, circulating kidney injury molecule-1 (KIM-1) and serum glycopeptides^8–11,14,17,19–22^. Further investigation is warranted to identify tumor and (or) stromal biologic features that may define susceptible patient populations.

Here we report the final clinical data after 5 years of follow-up from KEYNOTE-426 and the results of a prespecified exploratory biomarker analysis that was conducted to determine whether molecular determinants relevant to the underlying disease biology are associated with clinical outcomes (ORR, PFS and OS) for pembrolizumab plus axitinib and for sunitinib in participants with advanced clear cell RCC.

## Results

### Participants

**Fig. 1:**
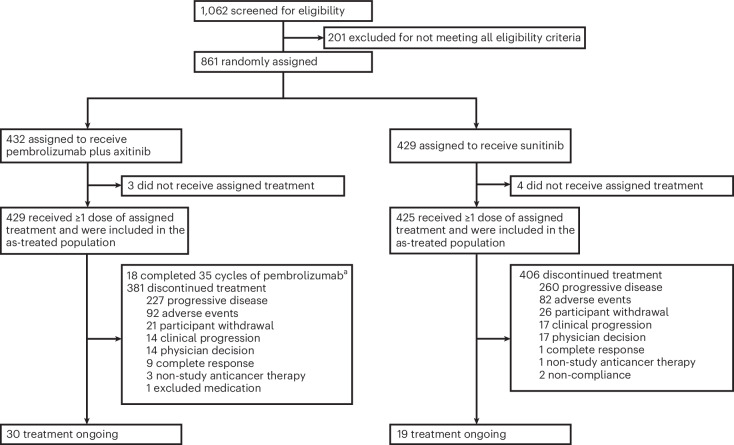
CONSORT diagram. ^a^Completed pembrolizumab treatment after discontinuing axitinib.

Between 24 October 2016 and 24 January 2018, 861 participants were randomly assigned to receive either pembrolizumab plus axitinib (*n* = 432) or sunitinib monotherapy (*n* = 429) (Fig. 1). As of the data cutoff date, the median follow-up (defined as time from randomization to the database cutoff date for this exploratory analysis) was 67.2 months (range, 60.0–75.0 months). Baseline demographics and characteristics are shown in Table 1. A total of 429 participants in the pembrolizumab plus axitinib arm and 425 participants in the sunitinib arm received at least one dose of the assigned study treatment. At the time of this analysis, 381 of 429 treated participants (88.8%) in the pembrolizumab plus axitinib arm and 406 of 425 treated participants (95.5%) in the sunitinib arm had permanently discontinued study treatment, most commonly due to radiographic progressive disease (pembrolizumab plus axitinib, *n* = 227 (52.9%); sunitinib, *n* = 260 (61.2%)) (Fig. 1). Thirty of 429 participants (7.0%) in the pembrolizumab plus axitinib arm and 19 of 425 participants (4.5%) in the sunitinib arm remained on treatment.

**Table 1:** Baseline characteristics in the intention-to-treat population

	Pembrolizumab plus axitinib *n* = 432	Sunitinib *n* = 429
Age, median (range), years	62.0 (30–89)	61.0 (26–90)
<65 years	260 (60.2)	278 (64.8)
Sex		
Male	308 (71.3)	320 (74.6)
Female	124 (28.7)	109 (25.4)
Region of enrollment		
North America	104 (24.1)	103 (24.0)
Western Europe	106 (24.5)	104 (24.2)
Rest of the world	222 (51.4)	222 (51.7)
IMDC risk group		
Favorable	138 (31.9)	131 (30.5)
Intermediate	238 (55.1)	246 (57.3)
Poor	56 (13.0)	52 (12.1)
Sarcomatoid features		
Yes	51 (11.8)	54 (12.6)
No	234 (54.2)	239 (55.7)
Unknown or missing	147 (34.0)	136 (31.7)
PD-L1 CPS^a^		
≥1	243 (56.3)	254 (59.2)
<1	167 (38.7)	158 (36.8)
Missing or unknown	22 (5.1)	17 (4.0)
No. of organs with metastases		
1	114 (26.4)	96 (22.4)
≥2	315 (72.9)	331 (77.2)
Missing	3 (0.7)	2 (0.5)
Most common sites of metastasis		
Lung	312 (72.2)	309 (72.0)
Lymph node	199 (46.1)	197 (45.9)
Bone	103 (23.8)	103 (24.0)
Adrenal gland	67 (15.5)	76 (17.7)
Liver	66 (15.3)	71 (16.6)
Previous radiotherapy	41 (9.5)	40 (9.3)
Previous nephrectomy	357 (82.6)	358 (83.4)

Data are *n* (%) unless otherwise noted.

^a^PD-L1 expression was centrally determined using the PD-L1 IHC 22C3 pharmDx (Agilent Technologies). CPS was calculated as the number of PD-L1-staining cells (tumor cells, lymphocytes and macrophages) divided by the total number of viable tumor cells, multiplied by 100.

Among participants who discontinued study treatment, 237 of 381 (62.2%) in the pembrolizumab plus axitinib arm and 300 of 406 (73.9%) in the sunitinib arm received subsequent systemic anticancer therapy, most commonly a VEGFR inhibitor in the pembrolizumab plus axitinib arm (206/237 (86.9%)) and a PD-L1 inhibitor in the sunitinib arm (240/300 (80.0%)) (Supplementary Table 1).

### Efficacy outcomes

**Fig. 2:**
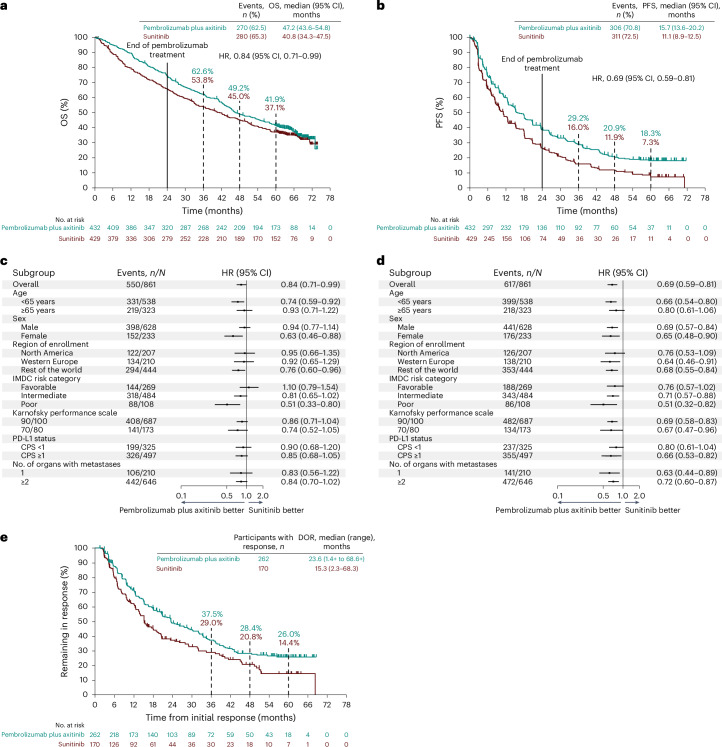
OS, PFS and DOR in the intention-to-treat population. **a**, Kaplan–Meier estimates of OS. **b**, Kaplan–Meier estimates of PFS. **c**, OS by subgroups. **d**, PFS by subgroups. **e**, Kaplan–Meier estimates of DOR in participants with a confirmed objective response. In **a**, **b** and **e**, tick marks represent censored data. In **c** and **d**, shaded squares correspond to the hazard ratios (HRs), and the error bars (horizontal lines) correspond to the 95% confidence intervals (CIs).

By the data cutoff date, 550 participants in the intention-to-treat population had died, including 270 of 432 participants (62.5%) in the pembrolizumab plus axitinib arm and 280 of 429 participants (65.3%) in the sunitinib arm. The median OS was 47.2 months in the pembrolizumab plus axitinib arm and 40.8 months in the sunitinib arm (hazard ratio: 0.84; 95% confidence interval: 0.71–0.99) (Fig. 2a). Median PFS was 15.7 months in the pembrolizumab plus axitinib arm and 11.1 months in the sunitinib arm (hazard ratio: 0.69; 95% confidence interval: 0.59–0.81) (Fig. 2b). Consistent OS and PFS benefits with pembrolizumab plus axitinib compared to sunitinib were seen across subgroups, including International Metastatic Renal Cell Carcinoma Database Consortium (IMDC) risk favorable and intermediate-risk and poor-risk categories and PD-L1 combined positive score (CPS) cutoff values of <1 and ≥1 (Fig. 2c,d).

Confirmed ORR was 60.6% (50 complete responses (11.6%), 212 partial responses (49.1%)) in the pembrolizumab plus axitinib arm and 39.6% (17 complete responses (4.0%), 153 partial responses (35.7%)) in the sunitinib arm (Extended Data Table 1). The median duration of response (DOR) was 23.6 months (range, 1.4+ months to 68.6+ months) in the pembrolizumab plus axitinib arm and 15.3 months (range, 2.3–68.3 months) in the sunitinib arm (Fig. 2e). The estimated percentage of participants with an objective response who would have an ongoing response at 60 months was 26.0% in the pembrolizumab plus axitinib arm and 14.4% in the sunitinib arm.

In a post hoc analysis of participants who completed 35 cycles of pembrolizumab (*n* = 120) (Supplementary Table 2), the median OS was not reached (95% confidence interval: 70.6 months to not reached), and the estimated 48-month and 60-month OS rates were 81.7% and 70.7%, respectively (Supplementary Fig. 1a). The median PFS was 37.4 months (95% confidence interval: 32.3–43.7 months), and the estimated 48-month and 60-month PFS rates were 38.3% and 32.8%, respectively (Supplementary Fig. 1b). The confirmed ORR was 85.0% (102/120; 22 complete responses (18.3%), 80 partial responses (66.7%)) (Supplementary Table 2).

### Biomarker outcomes

#### Biomarker analysis population

Of 848 RNA sequencing samples, 797 passed quality control, resulting in a 94.0% success rate. Of the 775 whole-exome sequencing (WES) tumor samples with matched normal samples, 751 passed quality control, resulting in a 96.9% success rate. Of the samples that passed quality control, duplicates and samples from participants who did not receive study treatment were further excluded from the biomarker analysis population. Of 854 participants who received at least one dose of study treatment in the total KEYNOTE-426 population, 730 (85.5%) had evaluable RNA sequencing data, 698 (81.7%) had evaluable WES data and 816 (95.6%) had evaluable PD-L1 CPS data (Supplementary Table 3). Baseline characteristics in the evaluable RNA sequencing and WES analyses populations were well balanced between treatment arms and similar to those of the total study population (Table 1 and Supplementary Table 3). Consistent with the intention-to-treat population, OS and PFS favored pembrolizumab plus axitinib over sunitinib in the RNA sequencing and WES analyses populations (Supplementary Table 3).

#### Tcell_inf_GEP, angiogenesis signature and PD-L1 CPS

**Fig. 3:**
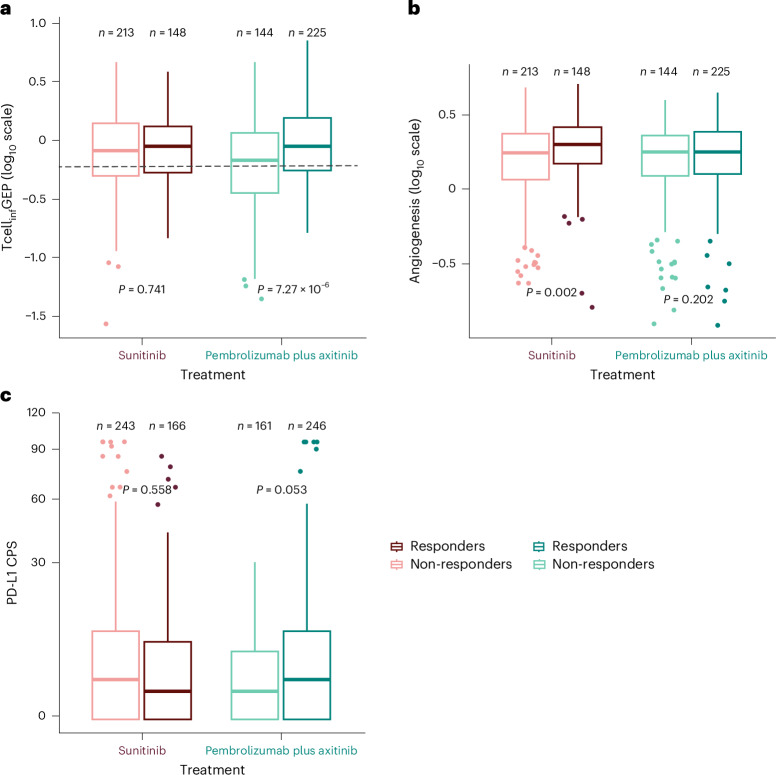
Participant-level distribution of select biomarker scores by response status and treatment arm. **a**, Tcell_inf_GEP (pembrolizumab plus axitinib, *n* = 369; sunitinib, *n* = 361). **b**, Angiogenesis signature (pembrolizumab plus axitinib, *n* = 369; sunitinib, *n* = 361). **c**, PD-L1 CPS (pembrolizumab plus axitinib, *n* = 407; sunitinib, *n* = 409). The center line corresponds to the median, and the box is delineated by the first and third quartiles. Whiskers extend to any points within 1.5 times the interquartile range, with points lying beyond identified individually as potential outliers. *P* values are nominal (one-sided for pembrolizumab plus axitinib and two-sided for sunitinib) and were derived using a logistic regression model, with adjustment for IMDC risk category. Significance was prespecified at α = 0.05.

**Extended Data Fig. 1:**
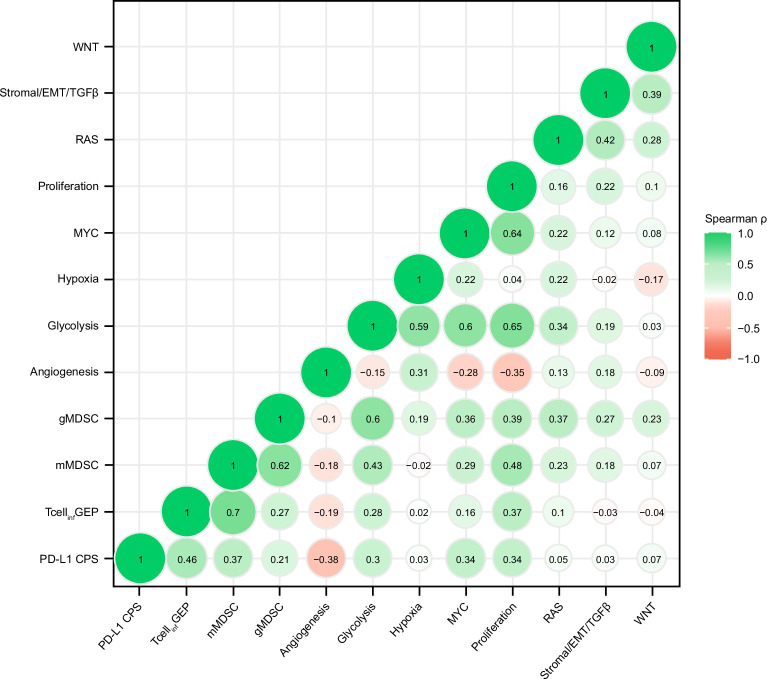
Correlogram plot of PD-L1 CPS, RNA sequencing signatures, and single genes. CPS, combined positive score; EMT, epithelial-to-mesenchymal transition; gMDSC, granulocytic myeloid-derived suppressor cell; mMDSC, monocytic myeloid-derived suppressor cell; PD-L1, programmed cell death ligand 1; Tcell_inf_GEP, T-cell–inflamed gene expression profile; TGF-β, transforming growth factor β.

Based on observed associations between biomarkers and clinical outcomes of pembrolizumab monotherapy in previous studies^23–28^, we examined whether an IFNγ-related 18-gene Tcell_inf_GEP, the angiogenesis signature and PD-L1 CPS were separately associated with clinical outcomes of pembrolizumab plus axitinib or sunitinib. In the pembrolizumab plus axitinib arm, higher Tcell_inf_GEP was associated with improved ORR (*P* < 0.0001), PFS (*P* < 0.0001) and OS (*P* = 0.002) (Table 2). In the sunitinib arm, no associations (*P* > 0.05) were observed between Tcell_inf_GEP and clinical outcomes (Table 2 and Fig. 3a). For the angiogenesis signature, a positive association was observed only with OS (*P* = 0.004) in the pembrolizumab plus axitinib arm, and there was a positive association with ORR (*P* = 0.002), PFS (*P* < 0.001) and OS (*P* < 0.001) in the sunitinib arm (Table 2 and Fig. 3b). The significance of Tcell_inf_GEP to clinical outcomes in the pembrolizumab plus axitinib arm and the significance of angiogenesis to clinical outcomes in the sunitinib arm remained the same in the joint Tcell_inf_GEP and angiogenesis model (Supplementary Table 4). PD-L1 CPS as a continuous variable (square root scale) was negatively associated with OS for sunitinib (*P* = 0.025). No association was observed between continuous PD-L1 CPS and clinical outcomes for pembrolizumab plus axitinib (*P* > 0.05) (Table 2 and Fig. 3c), suggesting that PD-L1 expression (as measured by CPS) is not a predictive marker of outcomes with pembrolizumab plus axitinib in this disease setting. However, PD-L1 CPS showed a moderate correlation with Tcell_inf_GEP (Spearman ρ = 0.46) (Extended Data Fig. 1), supportive of the respective roles of PD-L1 expression and Tcell_inf_GEP in biologically defining an inflamed tumor microenvironment.

**Table 2:** Within-arm association *P* values among gene expression signatures, PD-L1 CPS and molecular subtypes and clinical outcomes

	Pembrolizumab plus axitinib			Sunitinib		
	ORR	PFS	OS	ORR	PFS	OS
Gene expression signatures^**a**^						
Tcell_inf_GEP	**2.03** **×** **10**^**−6**^**(+)**	**1.41** **×** **10**^**−5**^**(+)**	**0.002 (+)**	0.741	0.464	0.547
Angiogenesis	0.202	0.244	**0.004 (+)**	**0.002 (+)**	**5.66** **×** **10**^**−4**^**(+)**	**1.69** **×** **10**^**−7**^**(+)**
Glycolysis	0.995	0.972	0.931	0.711	0.934	0.136
gMDSC	0.995	0.972	0.185	0.711	0.936	0.136
Hypoxia	0.243	0.265	0.404	**0.071 (+)**	0.979	**0.094(+)**
mMDSC	**0.058 (+)**	**0.039 (+)**	**0.057 (+)**	0.711	0.936	0.136
MYC	0.995	0.972	0.404	0.711	**0.017 (−)**	**1.50** **×** **10**^**−4**^**(−)**
Proliferation	0.995	0.972	0.314	0.684	0.269	**5.33** **×** **10**^**−4**^**(−)**
RAS	0.995	0.972	0.931	0.711	0.979	0.506
Stroma/EMT/TGFβ	0.995	0.972	0.931	0.711	0.979	0.479
WNT	0.995	0.150	0.931	0.711	0.979	0.526
Molecular subtypes^b^	0.243	0.265	0.202	0.711	0.979	**0.010**
PD-L1 CPS^c^	0.053	0.168	0.544	0.558	0.331	**0.025 (−)**

Association was evaluated using a logistic regression model (ORR) and a Cox proportional hazards regression model (PFS and OS), with adjustment for IMDC risk category. Bolded *P* values for Tcell_inf_GEP, angiogenesis signature and PD-L1 CPS indicate nominal statistical significance (α < 0.05); bolded *P* values for other gene expression signatures and molecular subtype indicate multiplicity-adjusted (Hochberg step-up procedure; tested as one family of 10 hypotheses within each treatment arm) statistical significance (α < 0.10). A ‘+’ or ‘−’ indicates that the observed association is positive or negative, respectively. In the pembrolizumab plus axitinib arm, a positive association (one-tailed test) was hypothesized for Tcell_inf_GEP and PD-L1 CPS; negative associations (one-tailed test) were hypothesized for glycolysis, proliferation, RAS and stroma/EMT/TGFβ; and non-zero associations (two-tailed test) were hypothesized for the remaining gene expression signatures and molecular subtype. In the sunitinib arm, a positive association (one-tailed test) was hypothesized for angiogenesis signature; negative associations (one-tailed test) were hypothesized for gMDSC, glycolysis, MYC, proliferation and RAS; and non-zero associations (two-tailed test) were hypothesized for the remaining gene expression signatures, PD-L1 CPS and molecular subtype. ^a^Included 369 participants in the pembrolizumab plus axitinib arm and 361 participants in the sunitinib arm. ^b^Likelihood ratio test was performed for molecular subtype by comparing the full model (with molecular subtype) with the reduced model (without molecular subtype). The analysis population included 369 participants in the pembrolizumab plus axitinib arm and 361 participants in the sunitinib arm. ^c^Association test for PD-L1 CPS was performed using the square root scale. The PD-L1 CPS analysis population included 407 participants in the pembrolizumab plus axitinib arm and 409 participants in the sunitinib arm.

**Fig. 4:**
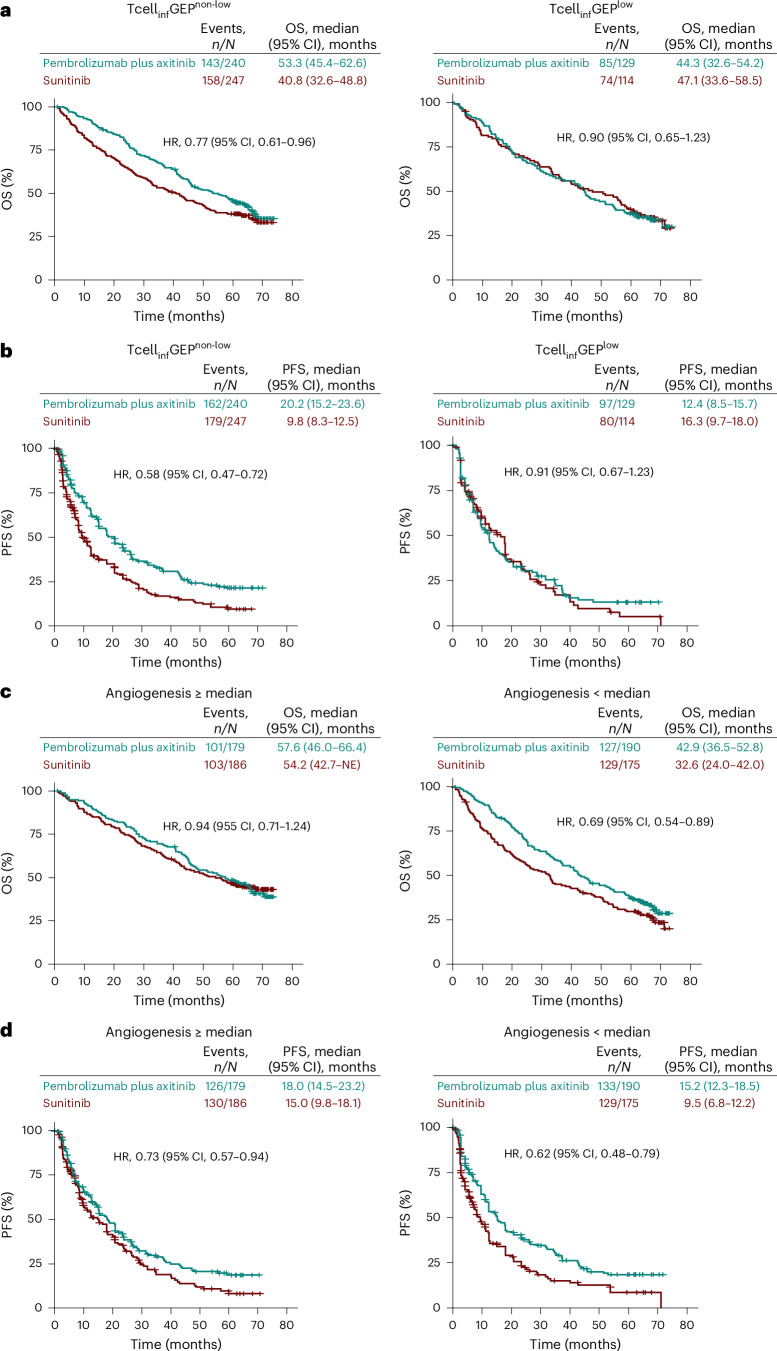
Kaplan–Meier estimates of survival by Tcell_inf_GEP or angiogenesis signature cutoff. **a**, OS by Tcell_inf_GEP cutoff. **b**, PFS by Tcell_inf_GEP cutoff. **c**, OS by angiogenesis signature cutoff. **d**, PFS by angiogenesis signature cutoff. Tick marks represent censored data. CI, confidence interval; HR, hazard ratio.

When assessing Tcell_inf_GEP using a prespecified cutoff of the first tertile, improved OS and PFS for pembrolizumab plus axitinib compared to sunitinib was observed in the Tcell_inf_GEP^non-low^ subgroup (OS hazard ratio: 0.77 (95% confidence interval: 0.61–0.96); PFS hazard ratio: 0.58 (95% confidence interval: 0.47–0.72)) (Fig. 4a,b). When assessing angiogenesis signature using a cutoff of ≥ or < the median, PFS favored pembrolizumab plus axitinib over sunitinib in the ≥ median subgroup (hazard ratio: 0.73 (95% confidence interval: 0.57–0.94)) (Fig. 4c,d). Notably, OS and PFS more strongly favored pembrolizumab plus axitinib over sunitinib in the < median subgroup (OS hazard ratio: 0.69 (95% confidence interval, 0.54–0.89); PFS hazard ratio: 0.62 (95% confidence interval: 0.48–0.79)) (Fig. 4c,d).

#### Other gene expression signatures and molecular subtype

We evaluated other gene expression signatures and their association with clinical outcomes by hypothesis testing within each treatment arm. In the pembrolizumab plus axitinib arm, the monocytic myeloid-derived suppressor cell (mMDSC) signature was positively associated with ORR (*P* = 0.058), PFS (*P* = 0.039) and OS (*P* = 0.057) (Table 2). In the sunitinib arm, the hypoxia signature was positively associated with ORR and OS (*P* = 0.071 and *P* = 0.094, respectively); the MYC signature was negatively associated with PFS and OS (*P* = 0.017 and *P* < 0.001, respectively); and the proliferation signature was negatively associated with OS (*P* < 0.001). When evaluating the independence of the gene signatures with the angiogenesis signature, the mMDSC signature was significantly associated with improved ORR (*P* = 0.006), PFS (*P* = 0.002) and OS (*P* = 0.004) in the pembrolizumab plus axitinib arm (Supplementary Table 4). After adjusting for the angiogenesis signature in the sunitinib arm, the hypoxia signature was no longer significantly associated with any clinical outcome; the MYC signature was negatively associated with PFS (*P* = 0.039) and OS (*P* = 0.016); and the proliferation signature was negatively associated with OS (*P* = 0.043). These associations were weaker compared to those without adjustment, which can be partially explained by the correlations among the signatures with the angiogenesis signature. Among all samples analyzed, there was a modest positive correlation (ρ = 0.31) (Extended Data Fig. 1) between the hypoxia and angiogenesis signatures, whereas there was a modestly negative correlation for the MYC and proliferation signatures with the angiogenesis signature (ρ = 0.28 and ρ = 0.35, respectively) (Extended Data Fig. 1).

Given that Tcell_inf_GEP was reported to be predictive of response to pembrolizumab monotherapy in other settings^24,29^, we adjusted these gene expression signatures for Tcell_inf_GEP (in addition to IMDC risk category) to elucidate their additional predictive value for the pembrolizumab and axitinib combination (although adjustment was performed in both treatment arms). After adjusting for Tcell_inf_GEP in the pembrolizumab plus axitinib arm, no associations between clinical outcomes and mMDSC signature were observed, and the proliferation signature was negatively associated with OS (*P* = 0.007) (Extended Data Table 2). Notably, mMDSC was strongly positively correlated with Tcell_inf_GEP (ρ = 0.70) (Extended Data Fig. 1). The associations for other gene expression signatures within the sunitinib arm remained similar after adjusting for Tcell_inf_GEP. The hypoxia signature was positively associated with ORR and OS (*P* = 0.065 and *P* = 0.095, respectively); the MYC signature was negatively associated with PFS and OS (*P* = 0.019 and *P* < 0.001, respectively); and the proliferation signature was negatively associated with OS (*P* < 0.001).

**Extended Data Fig. 2:**
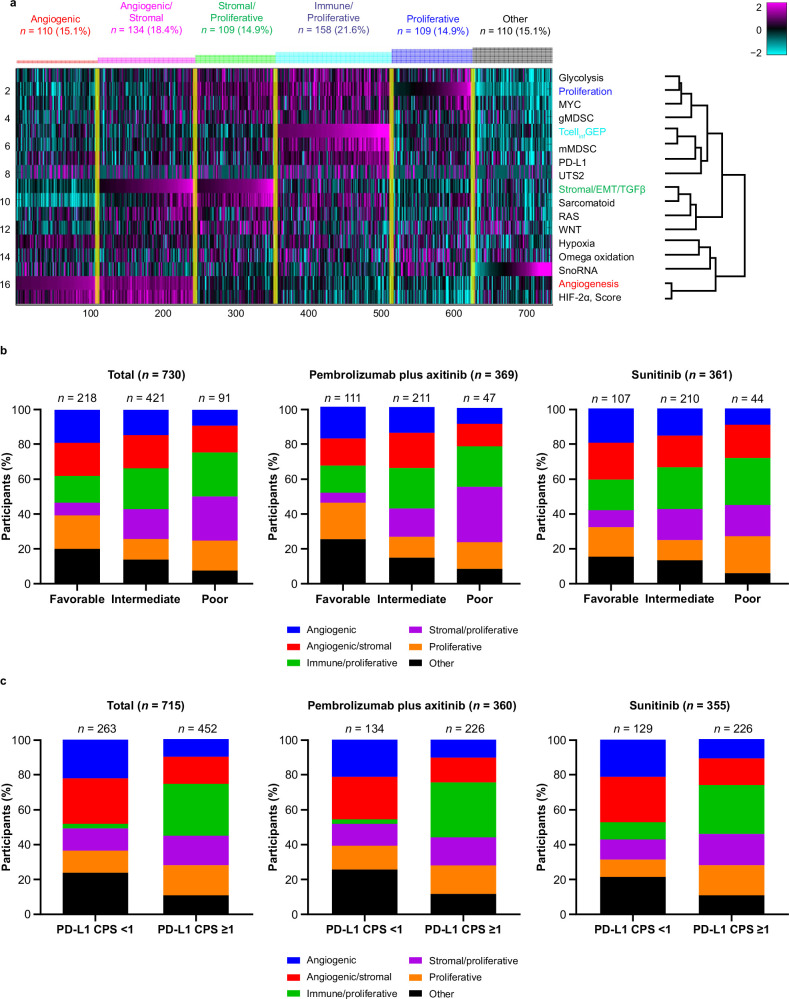
Clustering and distribution of molecular subtypes. **a**, Molecular subtype clustering. **b**, Distribution by IMDC risk categories. **c**, Distribution by tumor PD-L1 CPS. Molecular subtypes are based on transcriptomically defined clustering patterns identified in the IMmotion151 phase 3 study^8^. “Other” includes samples that could not be assigned to the angiogenic/stromal, angiogenic, immune/proliferative, proliferative, or stromal/proliferative subtype. CPS, combined positive score; EMT, epithelial-to-mesenchymal transition; gMDSC, granulocytic myeloid-derived suppressor cell; HIF-2α, hypoxia-inducible factor- α; IMDC, International Metastatic Renal Cell Carcinoma Database Consortium; mMDSC, monocytic myeloid-derived suppressor cell; PD-L1, programmed cell death ligand 1; Tcell_inf_GEP, T-cell–inflamed gene expression profile; TGF-β, transforming growth factor β.

We additionally sought to assign tumor samples to molecular subtypes according to the transcriptomically defined clustering in the phase 3 IMmotion151 trial^8^. We observed that 18.4% of the treated participants in the RNA sequencing population were angiogenic–stromal, 15.1% were angiogenic, 21.6% were immune–proliferative, 14.9% were proliferative and 14.9% were stromal–proliferative (Supplementary Table 5 and Extended Data Fig. 2a); 15.1% of participants in the total RNA sequencing population could not be assigned to one of these five subtypes and constituted a sixth ‘other’ subtype. Next, we evaluated the distribution of these molecular subtypes across IMDC risk categories and by PD-L1 status and observed enrichment of the immune–proliferative subtype in the IMDC intermediate-risk and poor-risk groups and in participants with tumors of PD-L1 CPS ≥ 1 (Extended Data Fig. 2b,c); the angiogenic–stromal subtype was enriched in participants with tumors of PD-L1 CPS < 1.

**Extended Data Fig. 3:**
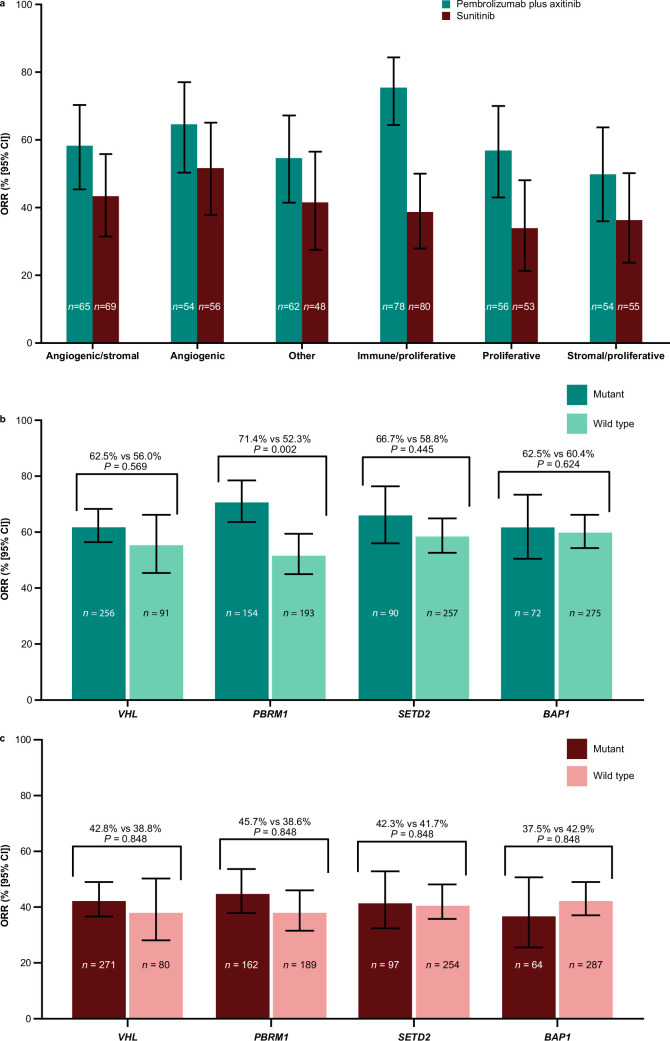
ORR by molecular subtypes and mutational status. **a**, ORR per transcriptomically defined clustering pattern identified in the IMmotion151 phase 3 study^8^ (pembrolizumab plus axitinib, *n* = 369; sunitinib, *n* = 361). **b**, ORR by mutational status in the pembrolizumab plus axitinib arm (*n* = 347). **c**, ORR by mutational status in the sunitinib arm (*n* = 351). In panel **a**, “Other” includes samples that could not be assigned to angiogenic/stromal, angiogenic, immune/proliferative, proliferative, or stromal/proliferative subtype. In panels **a-c**, bars represent ORR, derived by dividing the number of participants with a complete or partial response by the total number of patients in that subgroup, multiplied by 100. Error bars indicate 95% confidence intervals. *P* values shown in panels **b** and **c** are multiplicity-adjusted (one-sided for pembrolizumab plus axitinib and two-sided for sunitinib) and were derived using logistic regression model, with adjustment for IMDC risk category. Significance was prespecified at α = 0.10. *BAP1*, *BRCA1*-associated protein 1 gene; IMDC, International Metastatic Renal Cell Carcinoma Database Consortium; NS, not significant; ORR, objective response rate; *PBRM1*, polybromo-1 gene; *SETD2*, SET domain containing 2, histone lysine methyltransferase gene; *VHL*, von Lindau-Hippel tumor suppressor gene.

Testing of the association of molecular subtype and clinical outcomes showed an association with OS (*P* = 0.010) in the sunitinib arm (Table 2). After prespecified adjustment for Tcell_inf_GEP and the angiogenesis signature, no significant associations with clinical outcomes were observed in the pembrolizumab plus axitinib and sunitinib arms (*P* > 0.10) (Extended Data Table 2). Within the pembrolizumab plus axitinib arm, the ORR was lowest (50.0%) in the stromal–proliferative subtype and highest (75.6%) in the immune–proliferative subtype (Extended Data Fig. 3a). Within the sunitinib arm, the ORR was lowest (34.0%) in the proliferative subtype and highest (51.8%) in the angiogenic subtype.

#### Efficacy estimates by DNA mutational status

We examined the impact of mutations in genes with clinical relevance to RCC in other studies, including *VHL*, *SETD2* (SET domain containing 2, histone lysine methyltransferase), *PBRM1* and *BAP1* using WES^8,14,30^. In the pembrolizumab plus axitinib arm, no significant associations between DNA mutations and PFS or OS were observed (Extended Data Table 3). The *PBRM1* mutation was positively associated with the ORR within the pembrolizumab plus axitinib arm, with significantly higher rates in the *PBRM1* mutant than in the wild-type subgroup (71.4% versus 52.3%; *P* = 0.002) (Extended Data Table 3 and Extended Data Fig. 3b). The ORR with the pembrolizumab plus axitinib was similar between the mutant and the wild-type subgroups for *VHL*, *SETD2* and *BAP1* (Extended Data Fig. 3b). In the sunitinib arm, *VHL* and *PBRM1* mutations were associated with longer OS (*P* = 0.040 and *P* = 0.010, respectively), whereas *BAP1* mutation was associated with shorter OS (*P* = 0.019) (Extended Data Table 3). The *VHL*, *PBRM1* and *SETD2* mutations were not associated with PFS or ORR in the sunitinib arm (*P* > 0.10). The ORR with sunitinib was similar between the mutant and the wild-type subgroups for *VHL*, *PBRM1*, *SETD2* and *BAP1* (Extended Data Fig. 3c).

**Extended Data Fig. 4:**
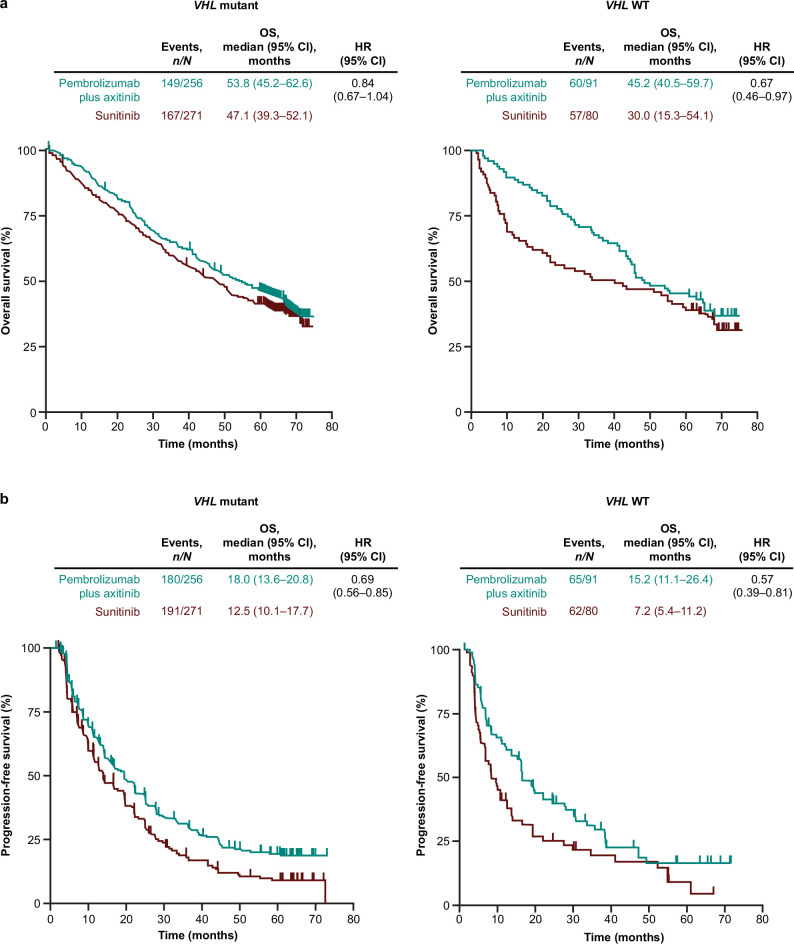
Kaplan-Meier estimates of survival by *VHL* mutational status. **a**, Overall survival. **b**, Progression-free survival. HR was estimated using a Cox proportional hazards model, with adjustment for IMDC risk category. Tick marks represent censored data. HR, hazard ratio; IMDC, International Metastatic Renal Cell Carcinoma Database Consortium; OS, overall survival; PFS, progression-free survival; *VHL*, von Lindau-Hippel tumor suppressor gene; WT, wild type.

**Extended Data Fig. 5:**
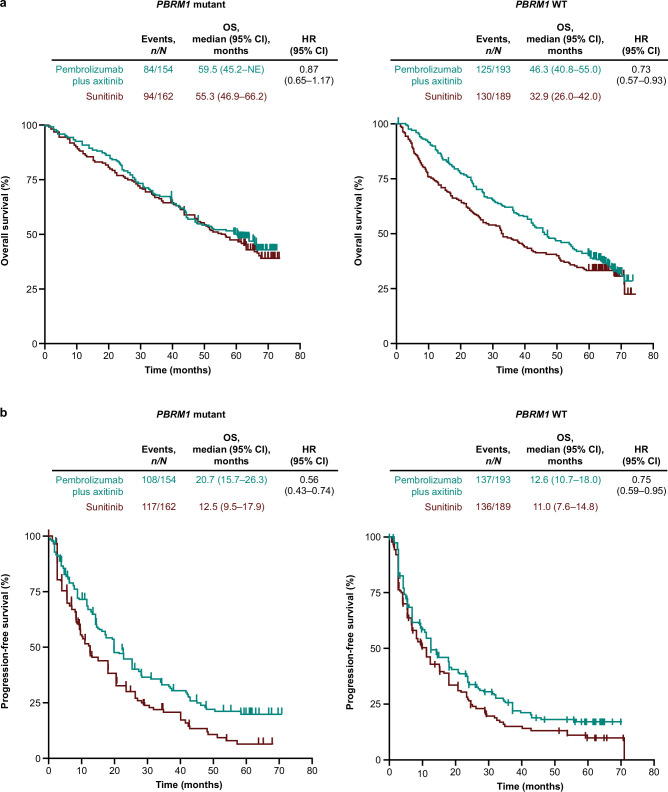
Kaplan-Meier estimates of survival by *PBRM1* mutational status. **a**, Overall survival. **b**, Progression-free survival. HR was estimated using the Cox proportional hazards model, with adjustment for IMDC risk category. HR, hazard ratio; IMDC, International Metastatic Renal Cell Carcinoma Database Consortium; NE, not evaluable; OS, overall survival; *PBRM1*, polybromo-1 gene; PFS, progression-free survival; WT, wild type.

**Extended Data Fig. 6:**
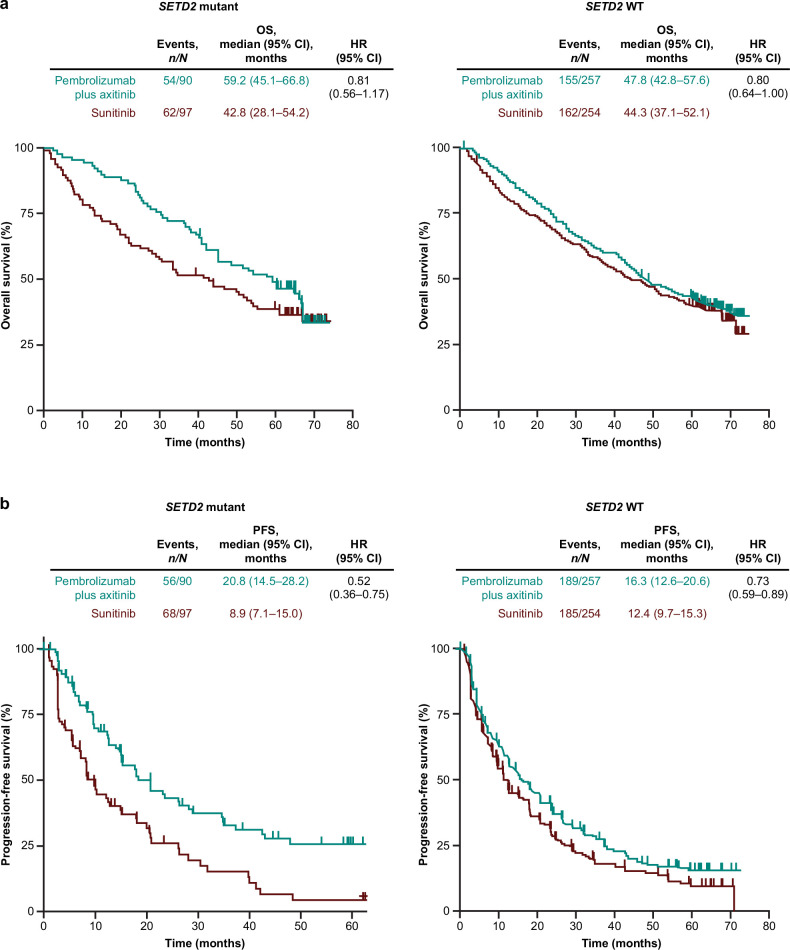
Kaplan-Meier estimates of survival by *SETD2* mutational status. **a**, Overall survival. **b**, Progression-free survival. HR was estimated using the Cox proportional hazards model, with adjustment for IMDC risk category. HR, hazard ratio; IMDC, International Metastatic Renal Cell Carcinoma Database Consortium; OS, overall survival; PFS, progression-free survival; *SETD2*, SET domain containing 2, histone lysine methyltransferase gene; WT, wild type.

**Extended Data Fig. 7:**
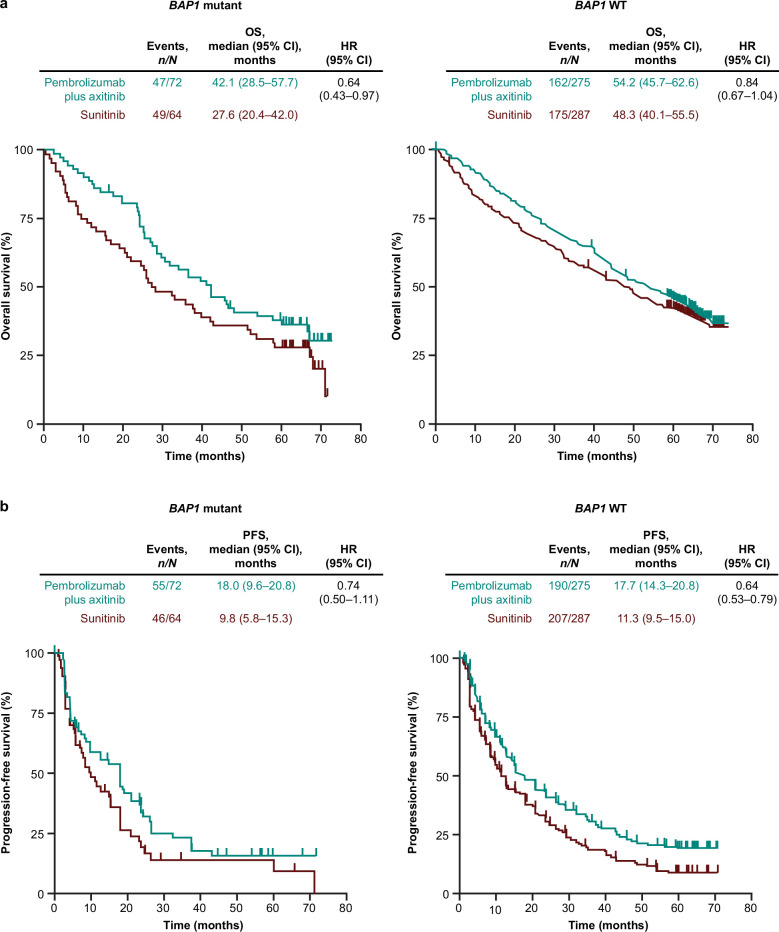
Kaplan-Meier estimates of survival by *BAP1* mutational status. **a**, Overall survival. **b**, Progression-free survival. HR was estimated using the Cox proportional hazards model, with adjustment for IMDC risk category. *BAP1*, *BRCA1*-associated protein 1 gene; HR, hazard ratio; IMDC, International Metastatic Renal Cell Carcinoma Database Consortium; OS, overall survival; PFS, progression-free survival; WT, wild type.

When assessing efficacy by DNA mutational status, OS and PFS directionally favored pembrolizumab plus axitinib over sunitinib, with various hazard ratios in the mutant and wild-type subgroups for *VHL*, *PBRM1*, *SETD2* and *BAP1* (Extended Data Figs. 4–7).

## Discussion

The final clinical follow-up analysis of KEYNOTE-426 showed sustained and durable clinical benefit of pembrolizumab plus axitinib compared to sunitinib^2,31,32^. In the prespecified exploratory biomarker analysis, significant associations between several genomic features and clinical outcomes with pembrolizumab plus axitinib or sunitinib were observed, which deepens understanding of RCC biology and potentially informs further advancement in treating patients with advanced clear cell RCC.

Higher Tcell_inf_GEP was associated with improved ORR, PFS and OS for pembrolizumab plus axitinib, but there was no association with clinical outcomes for sunitinib. This association with pembrolizumab plus axitinib was not unexpected given that the Tcell_inf_GEP comprises genes related to antigen presentation, adaptive immune resistance, cytolytic activity and chemokine expression^23,29^. The positive association between Tcell_inf_GEP and outcomes in the combination arm is consistent with previous reports for pembrolizumab monotherapy in the pan-tumor and specific tumor settings, including clear cell RCC managed with pembrolizumab monotherapy (KEYNOTE-427 cohort A) and with reports that showed associations between T cell inflammation-related genes and clinical outcomes for avelumab plus axitinib (JAVELIN Renal 101 trial) and atezolizumab plus bevacizumab (IMmotion150/151 trial)^23–29^. However, in the phase 3 CLEAR/KEYNOTE-581 trial of participants with advanced clear cell RCC, Tcell_inf_GEP was not associated with ORR and PFS for lenvatinib plus pembrolizumab but was associated with ORR for sunitinib^33^. In the phase 3 CheckMate 9ER trial of participants with advanced clear cell RCC, several gene expression signatures, including IFNγ, were not predictive of clinical outcomes of nivolumab plus cabozantinib^19^. The precise reasons for differences among the associations of immune signatures and outcomes across trials are not entirely clear but could include differences in the TKI partner, the number of evaluable samples and the duration of follow-up. The strength of the association between Tcell_inf_GEP and response seems to be greater with the pembrolizumab and axitinib combination than with pembrolizumab monotherapy in specific tumor types (including clear cell RCC in the KEYNOTE-427 trial)^23,25,27–29^, suggesting a potential positive interaction between the TKI and the PD-1 inhibitor. Preclinical models have shown that TKIs exert immune-modulatory effects in the tumor microenvironment by enhancing tumor cell sensitivity to immune-cell-mediated lysis through an alteration in the tumor cell phenotype and by altering the frequency or function of immune cell subsets in the periphery or the tumor microenvironment, thus promoting more productive immune interactions^34–36^. The hypothesis of a positive interaction between axitinib and pembrolizumab is further supported by the lack of significant associations between Tcell_inf_GEP and clinical outcomes for sunitinib, which suggests that the Tcell_inf_GEP is not a predictor of response to sunitinib as monotherapy. However, axitinib and sunitinib are different TKIs.

The strong positive association between angiogenesis signature and all clinical outcomes for sunitinib is consistent with previous reports from the phase 3 COMPARZ (first-line pazopanib versus sunitinib) and IMmotion151 (first-line atezolizumab plus bevacizumab versus sunitinib) studies of participants with advanced clear cell RCC, supporting the present findings^8,10,14^. Because TKI monotherapy is not commonly used as first-line therapy, these findings have limited clinical applicability. Whether this association would be seen in a refractory setting in which TKIs are commonly used requires further investigation.

Previous analyses of the IMmotion151 trial identified seven molecular clusters related to differential clinical outcomes with first-line atezolizumab plus bevacizumab compared to sunitinib across molecular subtypes^8,10^. Survival outcomes were poorer for participants with tumors classified within the angiogenic cluster who were given atezolizumab plus bevacizumab than for those given sunitinib (OS hazard ratio: 1.32)^10^. In the present analysis, results of assessment of the associations within arms showed a positive association of the angiogenesis signature with OS, and the OS hazard ratio for pembrolizumab plus axitinib compared to sunitinib in the high (≥ median) angiogenesis subgroup was 0.94. An understanding of the different antiangiogenic effects among different TKIs and between TKIs and bevacizumab as related to the association with an angiogenic gene signature requires further investigation. In the phase 3 JAVELIN Renal 101 trial, participants with advanced or metastatic RCC in clusters 1 (angiogenic–stromal), 3 (complement–oxidation) and 4 (T-effector–proliferative) treated with avelumab plus axitinib tended to have improved PFS compared to sunitinib-treated participants^11^. In the present analysis of the KEYNOTE-426 trial, the highest ORR for pembrolizumab plus axitinib was observed in the immune–proliferative cluster, as expected given that this cluster comprises tumors that are angiogenesis poor but highly immunogenic, with highest infiltration in immune cell subsets (CD8^+^, CD4^+^ and regulatory T cells, B cells, macrophages and dendritic cells)^8,10^.

Genomic features, such as loss-of-function mutations in *VHL*, *PBRM1*, *SETD2* and *BAP1*, have been evaluated to determine their association with clinical outcomes of systemic therapies in patients with advanced clear cell RCC; however, data are often conflicting^8,12–18,30^. The relationship between clinical outcomes and *PBRM1* DNA alterations has attracted attention in the past. Although the pressent dataset showed that *PBRM1* mutation tended to be associated with improved outcomes, *PRRM1* mutation does not seem to be a reliable biomarker considering the totality of the data.

Although PD-L1 CPS has been positively associated with clinical outcomes of pembrolizumab monotherapy or pembrolizumab-based combination treatment in advanced clear cell RCC and other tumor types^25,28^, the lack of a significant association between PD-L1 CPS and clinical outcomes in the present analyses suggests that PD-L1 expression (as measured by CPS) is not a predictive marker of outcomes of pembrolizumab plus axitinib in this disease setting and should not be used in this clinical setting. Similarly, PD-L1 expression (tumor proportion score ≥ 1%) was not associated with clinical outcomes of nivolumab plus cabozantinib in the CheckMate 9ER trial^19^. Notably, Tcell_inf_GEP, which was positively associated with clinical outcomes within the pembrolizumab plus axitinib arm, was moderately correlated with PD-L1 CPS (this correlation is not unexpected given that the Tcell_inf_GEP includes mRNA expressions for PD-L1)^29^. These data suggest the respective roles of Tcell_inf_GEP and PD-L1 CPS in biologically defining an inflamed tumor microenvironment, but their independent contribution as predictive biomarkers of immunotherapy may be therapy specific and tumor type specific^24,26,29^.

These data have both clinical and biomarker relevance in the treatment choice for patients with advanced clear cell RCC. First, with longer follow-up of patients in the KEYNOTE-426 trial and other phase 3 trials of a VEGF-TKI plus PD-1 inhibitor (CheckMate 9ER (cabozantinib plus nivolumab) and CLEAR/KEYNOTE-581 (lenvatinib plus pembrolizumab)), as well as more mature data for the cytotoxic T-lymphocyte-associated protein 4 (CTLA-4) inhibitor ipilimumab plus nivolumab (CheckMate 214), all four treatment approaches remain reasonable, supported by a significant survival advantage^37–39^. Second, employing the IMDC risk score to select therapy appears increasingly flawed; it remains a useful prognostic tool based on clinical characteristics rather than tissue-based biomarkers. Biomarker data from this and previous trials show some consistency but also many instances of inconsistency^8–11,14,19,20^. Although the signatures evaluated in this trial look promising, particularly for sunitinib, and other studies have shown potential clinical utility of molecular subsets and emerging biomarkers (for example, serum glycopeptides and circulating KIM-1)^8,10,11,20–22^, it is not currently possible to define a biomarker to select for combination regimens with either a specific VEGF-TKI plus PD-1 inhibitor or CTLA-4 inhibitor plus PD-1 inhibitor. Moving forward, prospective biomarker trials are ongoing, including those testing for PD-L1 expression and RNA signatures^30,40,41^.

A strength of the biomarker analysis is that the populations included a high proportion of the treated population in each arm; therefore, inferences drawn from these respective datasets are largely representative of the KEYNOTE-426 trial population. However, the prespecified exploratory biomarker analysis from the KEYNOTE-426 trial is limited by the small sample sizes of some of the subgroups and the lack of statistical power and (or) multiplicity adjustments for association analysis of some biomarkers, hindering definitive conclusions. Additionally, given the complex interplay of biological processes involved in the RCC tumor microenvironment^42^, the evaluation of each signature or gene individually most likely does not capture the potential joint effects of the biomarkers on clinical outcomes within each treatment arm. Furthermore, inter-trial differences in definitions for PD-L1 expression, different algorithms for clustering patient samples into molecular subtypes and different gene expression signatures evaluated limit comparative interpretations of the biomarker data. Lastly, because a VEGF-TKI was present in both treatment arms, the relative contribution of VEGF-TKI in the survival outcomes cannot be determined from a clinical and biomarker standpoint.

In conclusion, results of the present analysis showed sustained OS, PFS and ORR benefit of the use of pembrolizumab plus axitinib compared to sunitinib monotherapy. An extensive biomarker analysis adds to the increasing amount of information on biomarkers in patients treated with immunotherapy-based combinations. Although the analysis showed potential clinical utility of some RNA signatures in identifying patients who are likely to benefit the most from each treatment, additional correlative data and further prospective clinical investigations are needed to inform biomarker-directed treatment of patients with advanced or metastatic RCC who are being considered for combination treatment with antiangiogenic and PD-1 inhibitor therapies. Pembrolizumab plus axitinib is a first-line treatment option for patients with advanced RCC regardless of biomarker subtypes.

## Methods

### Inclusion and ethics

KEYNOTE-426 (NCT02853331) is a randomized, open-label, phase 3 trial conducted across 129 medical centers globally. The trial was conducted in accordance with the principles of Good Clinical Practice and was approved by the appropriate institutional review boards and regulatory agencies. Written informed consent was provided by all participants before enrollment.

### Trial design, participants and treatments

Details of the trial design and eligibility criteria were published previously^2,31^. In brief, eligible participants were adults with newly diagnosed stage IV or recurrent clear cell RCC who had not previously received systemic therapy for advanced disease. Participants had a Karnofsky Performance Scale score of 70% or higher at baseline, one or more measurable lesions per Response Evaluation Criteria in Solid Tumors version 1.1 (RECIST v1.1) as assessed by the investigator and a tumor sample available for biomarker assessment. Sex of participants was determined based on self-report.

Participants were randomly assigned in a 1:1 ratio to receive pembrolizumab 200 mg intravenously once every 3 weeks for up to 35 cycles (~2 years) plus axitinib 5 mg by mouth twice daily continuously or sunitinib 50 mg by mouth once daily for 4 weeks on and 2 weeks off, continuously. Randomization was stratified according to the IMDC risk group (favorable versus intermediate versus poor risk) and by geographic region (North America versus Western Europe versus rest of the world). Treatment was continued until disease progression, unacceptable toxicity or physician or participant decision to discontinue. In the pembrolizumab plus axitinib arm, if one drug was discontinued because of toxicity, the other drug could be continued.

### Outcomes

The dual primary endpoints of OS and PFS per RECIST v1.1 by blinded independent central review (BICR) and key secondary endpoint of ORR per RECIST v1.1 by BICR were met at the first interim analysis^2^. Therefore, the subsequent analyses of efficacy are exploratory.

The prespecified objectives of the exploratory biomarker analysis, defined in a statistical analysis plan, were as follows:

1. To assess whether an IFNγ-related 18-gene Tcell_inf_GEP and 10 other signatures (angiogenesis, glycolysis, granulocytic myeloid-derived suppressor cells (gMDSCs), hypoxia, mMDSCs, MYC, proliferation, RAS, stroma/EMT/TGFβ and WNT)^24^ are individually associated with clinical outcomes (ORR, OS and PFS) of pembrolizumab plus axitinib or sunitinib;
2. To assess whether prespecified molecular subtypes as categorical variables are separately associated with clinical outcomes of pembrolizumab plus axitinib or of sunitinib;
3. To assess whether continuous PD-L1 CPS is separately associated with clinical outcomes of pembrolizumab plus axitinib or of sunitinib; and
4. To assess whether mutation status of key RCC driver genes (*VHL*, *PBRM1*, *SETD2* and *BAP1*), as determined by WES, are separately associated with clinical outcomes of pembrolizumab plus axitinib or of sunitinib.

Estimation of PFS and OS hazard ratios for pembrolizumab plus axitinib compared to sunitinib was also performed by Tcell_inf_GEP subgroups based on a prespecified cutoff of the first tertile^23^; by angiogenesis signature subgroups defined by a prespecified cutoff of the median; and by mutational status of *VHL*, *PBRM1*, *SETD2* and *BAP1*.

### Assessments

Details on the assessment of efficacy outcomes were published previously and follow standard guidance by the US Food and Drug Administration (https://www.fda.gov/regulatory-information/search-fda-guidance-documents/clinical-trial-endpoints-approval-cancer-drugs-and-biologics)^2,31,43^. For the biomarker analysis, formalin-fixed, paraffin-embedded (FFPE) pretreatment tumor tissue samples collected at screening were used. PD-L1 expression was centrally determined using the PD-L1 IHC 22C3 pharmDx (Agilent Technologies). CPS was calculated as the number of PD-L1-staining cells (tumor cells, lymphocytes and macrophages) divided by the total number of viable tumor cells, multiplied by 100.

RNA sequencing was performed on an Illumina HiSeq by use of the TruSeq Access protocol. Raw reads were processed using a customized data analysis pipeline in OmicSoft Array Suite version 9 (Qiagen)^24^. In brief, the raw sequence reads were filtered for quality and subsequently aligned to the reference genome Human.B37.3 using OmicSoft Sequence Aligner^44^. After reference alignment, gene expression levels (raw read counts and fragments per kilobase of exon per million mapped fragments) were quantified using the RNA-Seq by Expectation Maximization (RSEM) algorithm^45^ with the gene model Ensembl.R75. The Tcell_inf_GEP is composed of 18 inflammatory genes related to antigen presentation, adaptive immune resistance, cytolytic activity and chemokine expression, including *CCL5*, *CD27*, *CD274* (PD-L1), *CD276* (B7-H3), *CD8A*, *CMKLR1*, *CXCL9*, *CXCR6*, *HLA–DQA1*, *HLA–DRB1*, *HLA-E*, *IDO1*, *LAG3*, *NKG7*, *PDCD1LG2* (PD-L2), *PSMB10*, *STAT1* and *TIGIT*^23,29^. The Tcell_inf_GEP score was calculated as the weighted sum of normalized expression values for the 18 genes. The Tcell_inf_GEP was established on the NanoString platform (NanoString Technologies), was evaluated across the pembrolizumab clinical development program and was predictive of response to pembrolizumab in both pan-tumor and histology-specific settings^23–25,27–29^. The 10 other signature scores (angiogenesis, glycolysis, gMDSCs, hypoxia, mMDSCs, MYC, proliferation, RAS, stroma/EMT/TGFβ and WNT) were calculated as the average of the genes (on the logarithmic scale) in each signature gene set, as previously described^24,29^.

Profiled tumor RNA sequencing samples were assigned to molecular subtypes according to the transcriptomically defined clustering in the phase 3 IMmotion151 trial of atezolizumab plus bevacizumab as first-line therapy compared to sunitinib in participants with advanced or metastatic RCC^8^. The molecular subtypes were assigned as follows. First, tumors with an angiogenesis consensus signature score above the upper tertile were assigned to the angiogenic group. Second, among tumors assigned to the angiogenic group, those that had a stroma/EMT/TGFβ consensus signature score above the median (evaluated over all samples) were assigned to the angiogenic–stromal group. Third, among the remaining samples, those that had a Tcell_inf_GEP score above the 75th percentile (evaluated over all samples) were assigned to the immune–proliferative group. Fourth, among the remaining (yet unassigned to the other groups) samples, those with a low proliferation consensus signature score (defined for which the proliferation score was below the lower tertile (evaluated over the not-already-assigned samples)) were assigned to the ‘other’ group. Finally, among the remaining samples, those for which a stroma/EMT/TGFβ score was higher than the median (evaluated over the remaining samples) were assigned to the stromal–proliferative group, whereas the other half was assigned to the proliferative group.

WES was performed on FFPE sections of pretreatment tumor samples and on matched normal (blood cell) samples using ACE Cancer Exome technology (Personalis), with average coverage of 200× (range, 13–475)^23,46^. WES reads were aligned to the Genome Reference Consortium Human Build 37 assembly by use of the Burrows–Wheeler Aligner MEM algorithm, followed by preprocessing steps that included duplicate marking, indel realignment and base recalibration using Picard (version 1.114; Broad Institute) and generation of analysis-ready binary alignment map files using Genome Analysis Toolkit (version 2; Broad Institute) analysis software. Thereafter, somatic single-nucleotide variant (SNV) calls were generated by comparing binary alignment map files from tumor and matched normal samples using default parameters from the MuTect method^47^. MuTect-called SNVs that were present in the Single Nucleotide Polymorphism Database (version 141; National Center for Biotechnology Information, https://www.ncbi.nlm.nih.gov/snp/) but not in the Catalogue of Somatic Mutations in Cancer (version 68; http://cancer.sanger.ac.uk) were filtered out^48^. SNVs with mutant reads of fewer than four in tumor samples were also eliminated. MuTect2 was further used to comprehensively characterize insertion or deletion–spliced mutations.

### Statistical analysis

We assessed efficacy in the intention-to-treat population, which included all randomly assigned participants, and followed guidelines published previously^2,31^. Because the trial outcome was previously defined as positive and the present analysis is exploratory, no formal hypothesis testing was performed in the present analysis. In the biomarker analysis population, we included all participants who received at least one dose of study treatment and had available PD-L1, RNA sequencing or WES data that passed quality control.

We used the Kaplan–Meier method to estimate OS, PFS and DOR in each treatment arm. To estimate the magnitude of the treatment difference (that is, hazard ratio) between the treatment arms, we used a stratified Cox proportional hazards model with the Efron method for handling ties. The stratification factors used for randomization were applied to the stratified Cox model. The stratified Miettinen and Nurminen method, with weights proportional to the stratum size, was used for comparison of ORR between the treatment arms. For ORR, 95% confidence intervals were based on the binomial exact confidence interval method for binomial data. Additionally, we assessed OS and PFS in protocol-prespecified subgroups based on participantsʼ baseline characteristics, including IMDC risk category and PD-L1 status. Post hoc analysis of efficacy was also performed for participants who completed 35 cycles of pembrolizumab. No formal hypothesis testing was conducted for the follow-up analysis of efficacy.

The biomarker analysis followed a statistical analysis plan written before merging of the clinical data with biomarker assessment, specifying where statistical testing would be used and what biomarker cutoffs defined the subgroups for treatment arm comparisons. The association between each biomarker and the clinical outcomes with pembrolizumab plus axitinib or sunitinib was assessed using logistic regression for ORR or a Cox proportional hazards regression model for OS and PFS. All models were adjusted by IMDC risk category, as prespecified in the statistical analysis plan. Statistical significance for associations between biomarkers and clinical outcomes (ORR, OS and PFS) was prespecified at α < 0.05 (without multiplicity adjustment) for Tcell_inf_GEP, the angiogenesis signature and PD-L1 CPS separately; at α < 0.10 after multiplicity adjustment (Hochberg step-up procedure; tested as one family of 10 hypotheses (before Tcell_inf_GEP adjustment) or nine hypotheses (after Tcell_inf_GEP adjustment) within each treatment arm) for the other signatures and molecular subtypes; and at α < 0.10 after multiplicity adjustment for DNA mutations, as prespecified in the statistical analysis plan. The direction of the hypothesis tests (that is, non-zero association (two-tailed test), positive association or negative association (one-tailed test)) for the Tcell_inf_GEP and other signatures was informed by an internal evaluation of published data from other trials, including the phase 3 JAVELIN Renal 101 trial of avelumab plus axitinib compared to sunitinib in participants with previously untreated advanced RCC^9,11^. The Spearman correlation was used to evaluate the relationship between pairs of RNA signatures and (or) biomarkers. Using prespecified cutoffs for the Tcell_inf_GEP (≥ first tertile (Tcell_inf_GEP^non-low^) and < first tertile (Tcell_inf_GEP^low^) as previously defined and validated using pan-tumor clinical data^23,49^) and angiogenesis signature (≥ median and < median; the choice of median as the cutoff was for illustrative purposes) and mutational status (mutant versus wild-type) for *VHL*, *PBRM1*, *SETD2* and *BAP1*, we performed descriptive subgroup analyses to estimate OS and PFS benefits of pembrolizumab plus axitinib compared to sunitinib and to assess the relative prognostic and predictive effects of the biomarkers. Statistical analyses were performed in SAS version 9.4 and R version 4.2.1 software. The data cutoff for this analysis was 23 January 2023.

### Reporting summary

Further information on research design is available in the Nature Portfolio Reporting Summary linked to this article.

## Online content

Any methods, additional references, Nature Portfolio reporting summaries, source data, extended data, supplementary information, acknowledgements, peer review information; details of author contributions and competing interests; and statements of data and code availability are available at 10.1038/s41591-025-03867-5.

## Supplementary information

Supplementary Information Supplementary Tables 1–5 and Supplementary Fig. 1

Reporting Summary

## Figures and Tables

**Extended Data Table 1:**
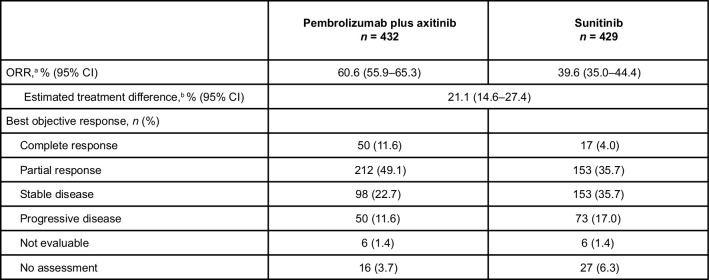
Summary of confirmed objective response per RECIST v1.1 by BICR in the intention-to-treat population

Percentages may not total 100 because of rounding. ^a^Includes participants who experienced complete response or partial response. ^b^Calculated based on the Miettinen and Nurminen method stratified by IMDC risk group (favorable versus intermediate versus poor) and geographic region (North America versus Western Europe versus rest of the world). ^c^Includes participants with postbaseline assessments available but not evaluable (that is, all postbaseline assessments with insufficient data for assessment of response per RECIST v1.1 or complete response, partial response or stable disease <6 weeks from randomization). ^d^Includes participants with no postbaseline assessment available for response evaluation.

**Extended Data Table 2:**
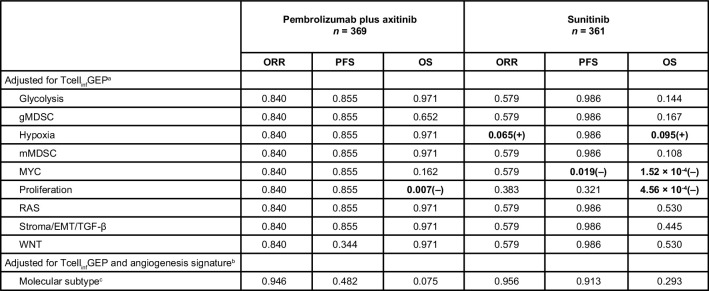
Within-arm association *P* values between gene expression signatures and molecular subtype and clinical outcomes, after adjustment for Tcell_inf_GEP and/or angiogenesis signature

Association was evaluated using a logistic regression model (ORR) and a Cox proportional hazards regression model (PFS and OS). In the pembrolizumab plus axitinib arm, a negative association (one-tailed test) was hypothesized for glycolysis, proliferation, RAS and stroma/EMT/TGFβ; non-zero associations (two-tailed test) were hypothesized for the remaining gene expression signatures and molecular subtype. In the sunitinib arm, negative associations (one-tailed test) were hypothesized for glycolysis, gMDSC, MYC, proliferation and RAS; non-zero associations (two-tailed test) were hypothesized for the remaining gene expression signatures and molecular subtype. A ‘+’ or ‘−’ indicates that the observed association is positive or negative, respectively. ^a^Bolded *P* values indicate nominal statistical significance (α < 0.05) for angiogenesis signature and multiplicity-adjusted (Hochberg step-up procedure; tested as one family of nine hypotheses within each treatment arm) statistical significance (α < 0.10) for other signatures; all models include additional covariates of IMDC risk category and Tcell_inf_GEP. ^b^Bolded *P* values indicate nominal statistical significance (α < 0.05); all models include additional covariates of IMDC risk category, Tcell_inf_GEP and angiogenesis signature. ^c^Likelihood ratio test was performed for molecular subtype by comparing the full model (with molecular subtype) to the reduced model (without molecular subtype).

**Extended Data Table 3:**
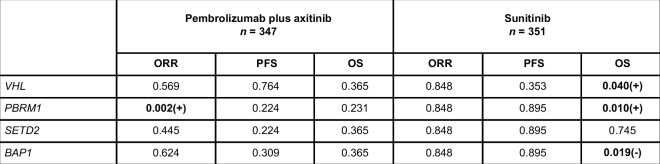
Within-arm association *P* values between DNA mutational status and clinical outcomes

Association was evaluated using a logistic regression model (ORR) and a Cox proportional hazards regression model (PFS and OS), with adjustment for IMDC risk category. Biomarkers were coded as 0 for wild-type status and 1 for mutant status. Bolded *P* values indicate multiplicity-adjusted statistical significance (α < 0.10). A ‘+’ or ‘−’ indicates that the observed association is positive or negative, respectively. In the pembrolizumab plus axitinib arm, a positive association (one-tailed test) was hypothesized for *SETD2*; non-zero associations (two-tailed test) were hypothesized for other biomarkers. In the axitinib arm, non-zero associations (two-tailed test) were hypothesized for all biomarkers.

## Data Availability

Merck Sharp & Dohme (MSD), a subsidiary of Merck & Co., is committed to providing qualified scientific researchers access to anonymized data and clinical study reports from the company’s clinical trials for the purpose of conducting legitimate scientific research. MSD is also obligated to protect the rights and privacy of trial participants and, as such, has a procedure in place for evaluating and fulfilling requests for sharing company clinical trial data with qualified external scientific researchers. The MSD data-sharing website (https://externaldatasharing-msd.com/) outlines the process and requirements for submitting a data request. Applications will be promptly assessed for completeness and policy compliance. Feasible requests will be reviewed by a committee of MSD subject matter experts to assess the scientific validity of the request and the qualifications of the requestors. In line with data privacy legislation, submitters of approved requests must enter into a standard data-sharing agreement with MSD before data access is granted. Data will be made available for request after product approval in the United States and the European Union or after product development is discontinued. Certain circumstances may prevent MSD from sharing requested data, including country-specific or region-specific regulations. If the request is declined, it will be communicated to the investigator. Access to genetic or exploratory biomarker data requires a detailed, hypothesis-driven statistical analysis plan that is collaboratively developed by the requestor and MSD subject matter experts; after approval of the statistical analysis plan and execution of a data-sharing agreement, MSD will either perform the proposed analyses and share the results with the requestor or will construct biomarker covariates and add them to a file with clinical data that is uploaded to an analysis portal so that the requestor can perform the proposed analyses.
