# Supplementary material for: Pembrolizumab plus axitinib versus sunitinib for advanced clear cell renal cell carcinoma: 5-year survival and biomarker analyses of the phase 3 KEYNOTE-426 trial
Source: Nat Med. 2025 Aug 1;31(10):3475–84. doi: 10.1038/s41591-025-03867-5 (PMC12532709; doi:10.1038/s41591-025-03867-5)
Supplement: Supplementary file 1 — Supplementary Tables 1–5 and Supplementary Fig. 1 [file 41591_2025_3867_MOESM1_ESM.pdf]

nature medicine

Supplementary information

https://doi.org/10.1038/s41591-025-03867-5

Pembrolizumab plus axitinib versus sunitinib for advanced clear cell renal cell carcinoma: 5-year survival and biomarker analyses of the phase 3 KEYNOTE-426 trial

In the format provided by the authors and unedited

Supplementary Materials

Supplementary Table 1. Subsequent systemic anticancer treatment after discontinuation of study treatment

	**Participants who discontinued study treatment**	
	**Pembrolizumab plus axitinib** *n* **= 381**	**Sunitinib** *n* **= 406**
**Received any subsequent systemic anticancer therapy**[a]	237/381 (62.2)	300 (73.9)
Any PD-(L)1 inhibitor	63/237 (26.6)	240/300 (80.0)
Any VEGF/VEGFR inhibitor	206/237 (86.9)	216/300 (72.0)
Any CTLA-4 inhibitor	10/237 (4.2)	23/300 (7.7)
Any cytotoxic chemotherapy	2/237 (0.8)	0/300 (0)
Any HIF-2α inhibitor	13/237 (0.5)	13/300 (4.3)
Any mTOR inhibitor	69/237 (29.1)	50/300 (16.7)
Other immunotherapies[b]	12/237 (5.1)	11/300 (3.7)
Other[c]	6/237 (2.5)	15/300 (5.0)
**Subsequent systemic therapy by line**		
≥1 subsequent line	236/237 (99.6)	300/300 (100)
≥2 subsequent lines	119/237 (50.2)	172/300 (57.3)
≥3 subsequent lines	60/237 (25.3)	94/300 (31.3)

Data are *n/N* (%). [a]Participants could have received more than 1 subsequent treatment. [b]Includes anti–CTLA-4/anti–PD-1 bispecific monoclonal antibody, dual CCR2 antagonist and CCR5 antagonist (unspecified), human anti–IL-27 antibody, ibrutinib, interferon, interferon alfa-2a, interferon alfa-2b, interferon gamma, and transduced autologous T cells. [c]Includes autogene cevumeran, erlotinib, glutaminase inhibitor, investigational drug, pexastimogene devacirepvec, rucaparib, savolitinib, and talazoparib. CTLA-4, cytotoxic T-lymphocyte–associated protein 4; HIF-2α, hypoxia-inducible factor 2 α; IL, interleukin; mTOR, mammalian target of rapamycin; PD-(L)1, programmed cell death

protein/ligand 1; VEGF, vascular endothelial growth factor; VEGFR, vascular endothelial growth factor receptor.

Supplementary Table 2 | Baseline characteristics and summary of confirmed objective response per RECIST v1.1 by BICR in participants who completed 35 cycles of pembrolizumab

	**Completed 35 cycles of pembrolizumab**<br>***n* = 120[a]**	**Total**<br>***N* = 432**
**Baseline characteristics**		
Age, median (range), years	61.5 (30–89)	62.0 (30–89)
<65 years	79 (65.8)	260 (60.2)
Sex		
Male	87 (72.5)	308 (71.3)
Female	33 (27.5)	124 (28.7)
Region of enrollment		
North America	23 (19.2)	104 (24.1)
Western Europe	23 (19.2)	106 (24.5)
Rest of the world	74 (61.7)	222 (51.4)
IMDC risk group		
Favorable	45 (37.5)	138 (31.9)
Intermediate	69 (57.5)	238 (55.1)
Poor	6 (5.0)	56 (13.0)
Sarcomatoid features		
Yes	17 (14.2)	51 (11.8)
No	65 (54.2)	234 (54.2)
Unknown or missing	38 (31.7)	147 (34.0)
PD-L1 CPS		
≥1	60 (50.0)	243 (56.3)

<1	54 (45.0)	167 (38.7)
Missing or unknown	6 (5.0)	22 (5.1)
No. of organs with metastases		
1	45 (37.5)	114 (26.4)
≥2	74 (61.7)	315 (72.9)
Missing	1 (0.8)	3 (0.7)
Most common sites of metastasis		
Lung	80 (66.7)	312 (72.2)
Lymph node	43 (35.8)	199 (46.1)
Bone	19 (15.8)	103 (23.8)
Adrenal gland	15 (12.5)	67 (15.5)
Liver	15 (12.5)	66 (15.3)
Previous radiotherapy	14 (11.7)	41 (9.5)
Previous nephrectomy	105 (87.5)	357 (82.6)
**Response**		
ORR,[b] % (95% CI)	85.0 (77.3–90.9)	60.6 (55.9–65.3)
Best objective response, *n* (%)		
Complete response	22 (18.3)	50 (11.6)
Partial response	80 (66.7)	212 (49.1)
Stable disease	14 (11.7)	98 (22.7)
Progressive disease	3 (2.5)	50 (11.6)
Not evaluable[c]	1 (0.8)	6 (1.4)
No assessment[d]	0 (0)	16 (3.7)

Data are *n* (%) unless otherwise noted. Percentages may not total 100 because of rounding. PD-L1 expression was centrally determined using the PD-L1 IHC 22C3 pharmDx (Agilent Technologies, Carpinteria, CA). CPS was calculated as the number of

PD-L1–staining cells (tumor cells, lymphocytes, and macrophages) divided by the total number of viable tumor cells, multiplied by 100. [a]A total of 27.8% (120/432) of participants assigned to the pembrolizumab plus axitinib arm. [b]Includes participants who experienced complete response or partial response. [c]Includes participants with postbaseline assessments available but not evaluable (ie, all postbaseline assessments with insufficient data for assessment of response per RECIST v1.1, or complete response, partial response, or stable disease <6 weeks from randomization). [d]Includes participants with no postbaseline assessment available for response evaluation. BICR, blinded independent central review; CPS, combined positive score; IMDC, International Metastatic Renal Cell Carcinoma Database Consortium; ORR, objective response rate; PD-L1, programmed cell death ligand 1; RECIST v1.1, Response Evaluation Criteria in Solid Tumors, version 1.1.

Supplementary Table 3 | Baseline characteristics and clinical outcomes in the evaluable RNA sequencing and WES populations

	**RNA sequencing**		**WES**	
	**Pembrolizumab plus axitinib**<br>***n* = 369**	**Sunitinib**<br>***n* = 361**	**Pembrolizuma b plus axitinib**<br>***n* = 347**	**Sunitinib**<br>***n* = 351**
**Baseline characteristics**				
Age, median (range), years	62 (30–87)	61 (26–88)	61 (30–87)	61 (26–88)
Sex				
Male	261 (70.7)	269 (74.5)	243 (70.0)	263 (74.9)
Female	108 (29.3)	92 (25.5)	104 (30.0)	88 (25.1)
Region of enrollment				
North America	94 (25.5)	91 (25.2)	87 (25.1)	84 (23.9)
Western Europe	100 (27.1)	94 (26)	95 (27.4)	96 (27.4)
Rest of the world	175 (47.4)	176 (48.8)	165 (47.6)	171 (48.7)
IMDC risk group				
Favorable	111 (30.1)	107 (29.6)	110 (31.7)	109 (31.1)
Intermediate	211 (57.2)	210 (58.2)	195 (56.2)	200 (57.0)
Poor	47 (12.7)	44 (12.2)	42 (12.1)	42 (12.0)
Sarcomatoid features				
Yes	46 (12.5)	47 (13.0)	44 (12.7)	47 (13.4)
No	202 (54.7)	202 (56.0)	188 (54.2)	199 (56.7)
Unknown or missing	121 (32.8)	112 (31.0)	115 (33.1)	105 (29.9)
PD-L1 CPS				
≥1	226 (61.2)	226 (62.6)	206 (59.4)	222 (63.2)

<1	134 (36.3)	129 (35.7)	133 (38.3)	120 (34.2)
Missing or unknown	9 (2.4)	6 (1.7)	8 (2.3)	9 (2.6)
**Clinical outcomes, HR (95% CI)**				
OS[a]	0.86 (0.72–1.04)		0.85 (0.70–1.03)	
PFS[a]	0.71 (0.60–0.84)		0.70 (0.59–0.84)	

Data are *n* (%) unless otherwise noted. [a]HR for pembrolizumab plus axitinib versus sunitinib. PD-L1 expression was centrally determined using the PD-L1 IHC 22C3 pharmDx (Agilent Technologies, Carpinteria, CA). CPS was calculated as the number of PD-L1–staining cells (tumor cells, lymphocytes, and macrophages) divided by the total number of viable tumor cells, multiplied by 100. CPS, combined positive score; HR, hazard ratio; IMDC, International Metastatic Renal Cell Carcinoma Database Consortium; OS, overall survival; PD-L1, programmed cell death ligand 1; PFS, progression-free survival; WES, whole exome sequencing.

Supplementary Table 4 | Within-arm association *P* values for the test of biomarker as independent predictors of clinical outcomes relative to the angiogenesis signature

	Pembrolizumab plus axitinib				Sunitinib			
	n	**ORR**	**PFS**	**OS**	***n***	**ORR**	**PFS**	**OS**
**$Tcell_{inf}GEP$**								
Angiogenesis	369	0.262	0.099	**0.003(+)**	361	**0.003(+)**	**0.002(+)**	**4.05×10^{-7}(+)**
$Tcell_{inf}GEP$	369	**1.78×10^{-5}(+)**	**1.25×10^{-5}(+)**	**0.003(+)**	361	0.410	0.863	0.955
**PD-L1 CPS**								
Angiogenesis	360	**0.025(+)**	**0.039(+)**	**0.001(+)**	355	**0.001(+)**	**7.28×10^{-5}(+)**	**3.20×10^{-7}(+)**
PD-L1 CPS	360	**0.032(+)**	0.096	0.180	355	0.513	0.920	0.493
**mMDSC**								
Angiogenesis	369	0.198	0.122	**0.002(+)**	361	**0.004(+)**	**0.002(+)**	**1.41×10^{-6}(+)**
mMDSC	369	**0.006(+)**	**0.002(+)**	**0.004(+)**	361	0.735	0.363	0.147
**Hypoxia**								
Angiogenesis	369	0.863	0.826	**0.022(+)**	361	0.057	**0.003(+)**	**2.19×10^{-5}(+)**
Hypoxia	369	0.076	0.084	0.781	361	0.131	0.833	0.915
**MYC**								

Angiogenesis	369	0.209	0.200	**0.008(+)**	361	**0.007(+)**	**0.018(+)**	**6.00 × 10^-4^(+)**
MYC	369	0.926	0.531	0.180	361	0.749	**0.039(-)**	**0.016(-)**
**Proliferation**								
Angiogenesis	369	0.149	0.122	**0.010(+)**	361	**0.009(+)**	**0.008(+)**	**3.58 × 10^-4^(+)**
Proliferation	369	0.119	0.161	0.280	361	0.774	0.455	**0.043(-)**

Association was evaluated using logistic regression model (ORR) and Cox proportional hazards regression model (PFS and OS). Nonzero associations (2-tailed test) were hypothesized. A + or − indicates the observed association is positive or negative, respectively. [a]Bolded *P* values indicate nominal statistical significance ($\alpha < 0.05$); the model includes additional covariates of IMDC risk.

CPS, combined positive score; IMDC, International Metastatic Renal Cell Carcinoma Database Consortium; mMDSC, monocytic myeloid-derived suppressor cells; ORR, objective response rate; OS, overall survival; PFS, progression-free survival; PD-L1, programmed cell death ligand 1; $Tcell_{inf}$GEP, T-cell–inflamed gene expression profile.

Supplementary Table 5 | Number of participants in each molecular subtype[a]

	**Pembrolizumab plus axitinib**<br>***n* = 369**	**Sunitinib**<br>***n* = 361**	**Total**<br>***N* = 730**
Angiogenic/stromal	65 (17.6)	69 (19.1)	134 (18.4)
Angiogenic	54 (14.6)	56 (15.5)	110 (15.1)
Immune/proliferative	78 (21.1)	80 (22.2)	158 (21.6)
Proliferative	56 (15.2)	53 (14.7)	109 (14.9)
Stromal/proliferative	54 (14.6)	55 (15.2)	109 (14.9)
Other[b]	62 (16.8)	48 (13.3)	110 (15.1)

Data are *n* (%). [a]Based on transcriptomically defined clustering pattern identified in the IMmotion151 phase 3 study[1]. [b]Includes participants who could not be assigned to the angiogenic/stromal, angiogenic, immune/proliferative, proliferative, or stromal/proliferative subtype.

Supplementary Fig. 1 | Kaplan-Meier estimates of survival in participants who completed 35 cycles of pembrolizumab. a, Overall survival. **b,** Progression-free survival. NR, not reached; OS, overall survival; PFS, progression-free survival.

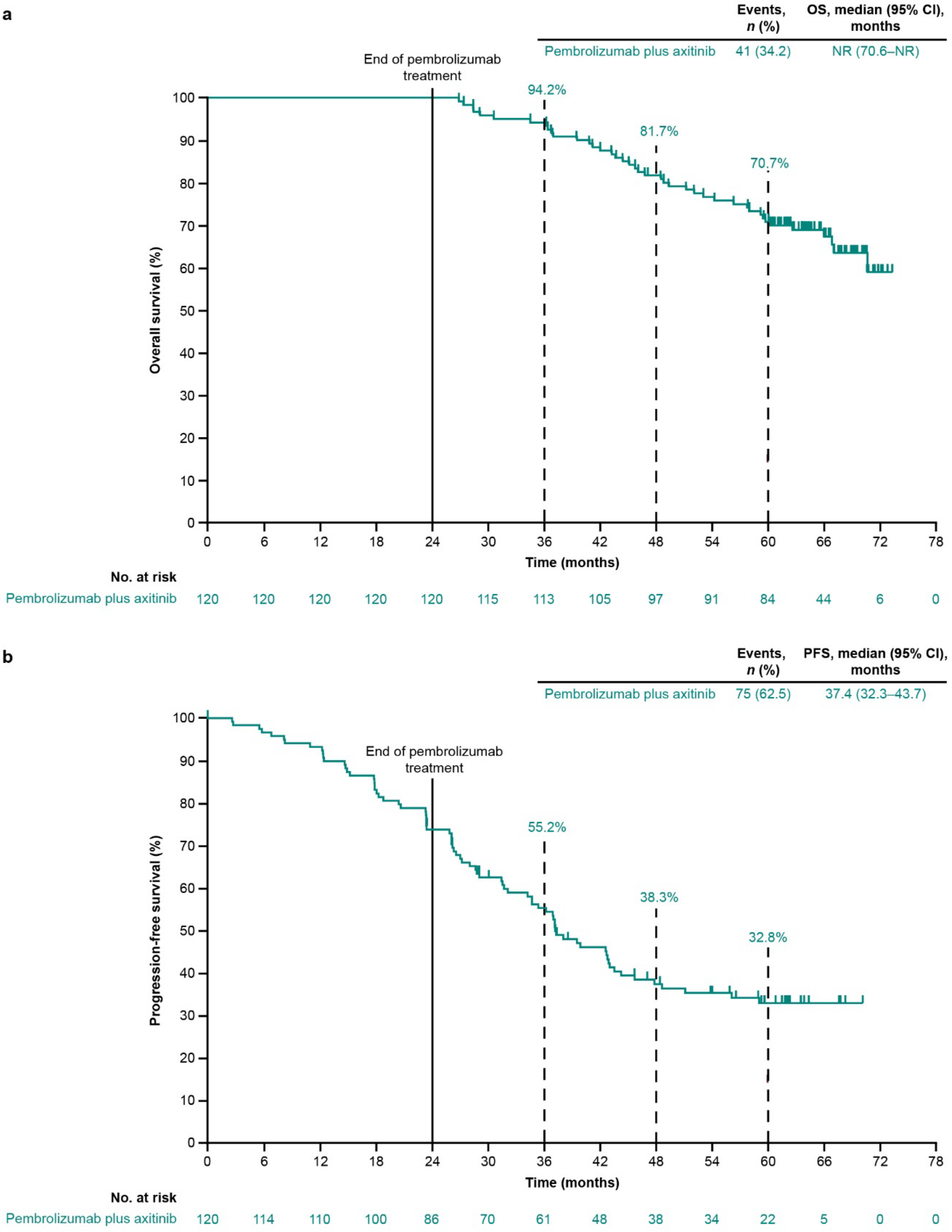

Supplementary reference

1. Motzer, R. J., et al. Molecular subsets in renal cancer determine outcome to checkpoint and angiogenesis blockade. *Cancer Cell* **38**, 803–817.e804 (2020).
