# Supplementary material for: Pembrolizumab plus axitinib versus sunitinib for advanced clear cell renal cell carcinoma: 5-year survival and biomarker analyses of the phase 3 KEYNOTE-426 trial
Source: Nat Med. 2025 Aug 1;31(10):3475–84. doi: 10.1038/s41591-025-03867-5 (PMC12532709; doi:10.1038/s41591-025-03867-5)
Supplement: Supplementary file 2 — Reporting Summary [file 41591_2025_3867_MOESM2_ESM.pdf]

nature portfolio

Corresponding author(s): Brian I. Rini

Last updated by author(s): May 12, 2025

Reporting Summary

Nature Portfolio wishes to improve the reproducibility of the work that we publish. This form provides structure for consistency and transparency in reporting. For further information on Nature Portfolio policies, see our Editorial Policies and the Editorial Policy Checklist.

Statistics

For all statistical analyses, confirm that the following items are present in the figure legend, table legend, main text, or Methods section.

n/a	Confirmed	
☐	☒	The exact sample size (*n*) for each experimental group/condition, given as a discrete number and unit of measurement
☒	☐	A statement on whether measurements were taken from distinct samples or whether the same sample was measured repeatedly
☐	☒	The statistical test(s) used AND whether they are one- or two-sided<br>*Only common tests should be described solely by name; describe more complex techniques in the Methods section.*
☐	☒	A description of all covariates tested
☐	☒	A description of any assumptions or corrections, such as tests of normality and adjustment for multiple comparisons
☐	☒	A full description of the statistical parameters including central tendency (e.g. means) or other basic estimates (e.g. regression coefficient) AND variation (e.g. standard deviation) or associated estimates of uncertainty (e.g. confidence intervals)
☐	☒	For null hypothesis testing, the test statistic (e.g. *F*, *t*, *r*) with confidence intervals, effect sizes, degrees of freedom and *P* value noted<br>*Give P values as exact values whenever suitable.*
☒	☐	For Bayesian analysis, information on the choice of priors and Markov chain Monte Carlo settings
☒	☐	For hierarchical and complex designs, identification of the appropriate level for tests and full reporting of outcomes
☒	☐	Estimates of effect sizes (e.g. Cohen's *d*, Pearson's *r*), indicating how they were calculated

Our web collection on statistics for biologists contains articles on many of the points above.

Software and code

Policy information about availability of computer code

Data collection	InForm 7.0 for clincial data analysis
Data analysis	SAS, version 9.4. and R version 4.2.1; OmicSoft Array Suite, version 9 (Qiagen, Hilden, Germany). Picard (version 1.114; Broad Institute, Cambridge, MA). The Genome Analysis Toolkit (version 2; Broad Institute. Single Nucleotide Polymorphism Database (v.141; National Center for Biotechnology Information, Bethesda, MD; https://www.ncbi.nlm.nih.gov/snp/). Burrows-Wheeler Aligner MEM algorithm. RNA-Seq by Expectation Maximization. OmicSoft Sequence Aligner. Catalogue of Somatic Mutations in Cancer (v.68; http://cancer.sanger.ac.uk)

For manuscripts utilizing custom algorithms or software that are central to the research but not yet described in published literature, software must be made available to editors and reviewers. We strongly encourage code deposition in a community repository (e.g. GitHub). See the Nature Portfolio guidelines for submitting code & software for further information.

Data

Policy information about availability of data

All manuscripts must include a data availability statement. This statement should provide the following information, where applicable:

- Accession codes, unique identifiers, or web links for publicly available datasets
- A description of any restrictions on data availability
- For clinical datasets or third party data, please ensure that the statement adheres to our policy

Merck Sharp & Dohme LLC, a subsidiary of Merck & Co., Inc., Rahway, NJ, USA (MSD), is committed to providing qualified scientific researchers access to

anonymized data and clinical study reports from the company's clinical trials for the purpose of conducting legitimate scientific research. MSD is also obligated to protect the rights and privacy of trial participants and, as such, has a procedure in place for evaluating and fulfilling requests for sharing company clinical trial data with qualified external scientific researchers. The MSD data sharing website (available at: https://externaldatasharing-msd.com/) outlines the process and requirements for submitting a data request. Applications will be promptly assessed for completeness and policy compliance. Feasible requests will be reviewed by a committee of MSD subject matter experts to assess the scientific validity of the request and the qualifications of the requestors. In line with data privacy legislation, submitters of approved requests must enter into a standard data-sharing agreement with MSD before data access is granted. Data will be made available for request after product approval in the United States and the European Union or after product development is discontinued. There are circumstances that may prevent MSD from sharing requested data, including country or region-specific regulations. If the request is declined, it will be communicated to the investigator. Access to genetic or exploratory biomarker data requires a detailed, hypothesis-driven statistical analysis plan that is collaboratively developed by the requestor and MSD subject matter experts; after approval of the statistical analysis plan and execution of a data-sharing agreement, MSD will either perform the proposed analyses and share the results with the requestor or will construct biomarker covariates and add them to a file with clinical data that is uploaded to an analysis portal so that the requestor can perform the proposed analyses.

Research involving human participants, their data, or biological material

Policy information about studies with human participants or human data. See also policy information about sex, gender (identity/presentation), and sexual orientation and race, ethnicity and racism.

Reporting on sex and gender	Data regarding sex are provided in the baseline demographics and disease characteristics table (Supplementary Table 1). Overall survival and progression-free survival outcomes by sex are reported in Figure 2. No individual-level data are presented.
Reporting on race, ethnicity, or other socially relevant groupings	No analyses of race, ethnicity, or other socially relevant groups were performed.
Population characteristics	The median age of the overall participant population was 62.0 years. The majority of participants in the overall study population were aged 65 or below (62.5%), male (72.9%), White (79.4%), and non Hispanic Or Latino (88.7%).
Recruitment	Patients who met the eligibility criteria were recruited by study investigators
Ethics oversight	The study was conducted in accordance with principles of Good Clinical Practice and was approved by the appropriate institutional review boards and regulatory agencies. Written informed consent was provided by all participants before enrollment.

Note that full information on the approval of the study protocol must also be provided in the manuscript.

Field-specific reporting

Please select the one below that is the best fit for your research. If you are not sure, read the appropriate sections before making your selection.

☒ Life sciences ☐ Behavioural & social sciences ☐ Ecological, evolutionary & environmental sciences

For a reference copy of the document with all sections, see nature.com/documents/nr-reporting-summary-flat.pdf

Life sciences study design

All studies must disclose on these points even when the disclosure is negative.

Sample size	Sample size was previously reported in the first interim analysis. We assessed efficacy in the intention-to-treat population, which included all randomly assigned participants, and followed guidelines published previously. 861 participants were randomly assigned to receive either pembrolizumab plus axitinib (n = 432) or sunitinib monotherapy (n = 429)
Data exclusions	We assessed efficacy in the intention-to-treat population, which included all randomly assigned participants. In the biomarker analysis population, we included all participants who received at least one dose of study treatment and had available PD-L1, RNA sequencing, or WES data that passed quality control.
Replication	This was a clinical study so no replication was attempted.
Randomization	Participants were randomly assigned in a 1:1 ratio to receive pembrolizumab 200 mg intravenously once every 3 weeks for up to 35 cycles (~2 years) plus axitinib 5 mg by mouth twice daily continuously or sunitinib 50 mg by mouth once daily for 4 weeks on and 2 weeks off, continuously. Randomization was done using an interactive voice response system or integrated web response system, and was stratified according to the International Metastatic Renal Cell Carcinoma Database Consortium (IMDC) risk group (favorable vs intermediate vs poor risk) and by geographic region (North America vs Western Europe vs rest of the world).
Blinding	This was an open-label study.

Reporting for specific materials, systems and methods

We require information from authors about some types of materials, experimental systems and methods used in many studies. Here, indicate whether each material, system or method listed is relevant to your study. If you are not sure if a list item applies to your research, read the appropriate section before selecting a response.

Materials & experimental systems

n/a	Involved in the study
☒	☐ Antibodies
☒	☐ Eukaryotic cell lines
☒	☐ Palaeontology and archaeology
☒	☐ Animals and other organisms
☐	☒ Clinical data
☒	☐ Dual use research of concern
☒	☐ Plants

Methods

n/a	Involved in the study
☒	☐ ChIP-seq
☒	☐ Flow cytometry
☒	☐ MRI-based neuroimaging

Clinical data

Policy information about clinical studies
All manuscripts should comply with the ICMJE guidelines for publication of clinical research and a completed CONSORT checklist must be included with all submissions.

Clinical trial registration

Clinicaltrials.gov, NCT02853331

Study protocol

Full protocol has been previously published

Data collection

Patients were enrolled from 129 centers (hospitals and cancer centers) globally between October 2016 and January 2018. For the biomarker analysis, formalin-fixed, paraffin-embedded pretreatment tumor tissue samples collected at screening were used. WES was performed on formalin-fixed paraffin-embedded sections of pretreatment tumor samples and on matched normal (blood cell) samples. RNA sequencing was performed on Illumina HiSeq (Illumina, Inc., San Diego, CA) by use of the TruSeq Access protocol.

Outcomes

The dual primary end points of OS and PFS per RECIST v1.1 by blinded independent central review (BICR) and key secondary end point of ORR per RECIST v1.1 by BICR.
The prespecified objectives of the exploratory biomarker analysis, defined in a statistical analysis plan, were as follows:
(1) To assess whether an IFN-γ–related 18-gene T-cell–inflamed gene expression profile (TcellinfGEP) and 10 other signatures (angiogenesis, glycolysis, granulocytic myeloid-derived suppressor cells [gMDSCs], hypoxia, monocytic myeloid-derived suppressor cells [mMDSCs], MYC, proliferation, RAS, stroma/epithelial-to-mesenchymal transition (EMT)/transforming growth factor β [TGF-β], and WNT)20 are individually associated with clinical outcomes (ORR, OS, and PFS) of pembrolizumab plus axitinib or sunitinib;
(2) To assess whether prespecified molecular subtypes as categorical variables are separately associated with clinical outcomes of pembrolizumab plus axitinib or of sunitinib;
(3) To assess whether continuous PD-L1 combined positive score (CPS) is separately associated with clinical outcomes of pembrolizumab plus axitinib or of sunitinib; and
(4) To assess whether mutation status of key RCC driver genes (von Hippel-Lindau tumor suppressor [VHL], PBRM1, SET domain containing 2, histone lysine methyltransferase [SETD2], and BRCA1-associated protein 1 [BAP1]), as determined by whole exome sequencing (WES), are separately associated with clinical outcomes of pembrolizumab plus axitinib or of sunitinib.

Plants

Seed stocks

NA

Novel plant genotypes

NA

Authentication

NA
